# Identification of burden hotspots and risk factors for cholera in India: An observational study

**DOI:** 10.1371/journal.pone.0183100

**Published:** 2017-08-24

**Authors:** Mohammad Ali, Sanjukta Sen Gupta, Nisha Arora, Pradeep Khasnobis, Srinivas Venkatesh, Dipika Sur, Gopinath B. Nair, David A. Sack, Nirmal K. Ganguly

**Affiliations:** 1 Department of International Health, Johns Hopkins Bloomberg School of Public Health, Baltimore, Maryland, United States of America; 2 Policy Center for Biomedical Research, Translational Health Science and Technology Institute, New Delhi, India; 3 National Center for Disease Control, New Delhi, India; 4 Indian Public Health Association, New Delhi, India; 5 World Health Organization, New Delhi, India; University of Miami, UNITED STATES

## Abstract

**Background:**

Even though cholera has existed for centuries and many parts of the country have sporadic, endemic and epidemic cholera, it is still an under-recognized health problem in India. A Cholera Expert Group in the country was established to gather evidence and to prepare a road map for control of cholera in India. This paper identifies cholera burden hotspots and factors associated with an increased risk of the disease.

**Methodology/Principle findings:**

We acquired district level data on cholera case reports of 2010–2015 from the Integrated Disease Surveillance Program. Socioeconomic characteristics and coverage of water and sanitation was obtained from the 2011 census. Spatial analysis was performed to identify cholera hotspots, and a zero-inflated Poisson regression was employed to identify the factors associated with cholera and predicted case count in the district. 27,615 cholera cases were reported during the 6-year period. Twenty-four of 36 states of India reported cholera during these years, and 13 states were classified as endemic. Of 641 districts, 78 districts in 15 states were identified as “hotspots” based on the reported cases. On the other hand, 111 districts in nine states were identified as “hotspots” from model-based predicted number of cases. The risk for cholera in a district was negatively associated with the coverage of literate persons, households using treated water source and owning mobile telephone, and positively associated with the coverage of poor sanitation and drainage conditions and urbanization level in the district.

**Conclusions/Significance:**

The study reaffirms that cholera continues to occur throughout a large part of India and identifies the burden hotspots and risk factors. Policymakers may use the findings of the article to develop a roadmap for prevention and control of cholera in India.

## Introduction

Cholera has existed in India for centuries, and is an important public health problem in several parts of the country [1]. Based on a ten year review from 1997 to 2006, 21 states reported cholera and 12 had multiple outbreaks [2]. During the 10-year period studied, the states having the highest number of reported outbreaks were West Bengal, Odisha, Maharashtra and Kerala [2]. These data illustrate that cholera occurs over a wider geographic area in India than is commonly perceived and is not restricted only to the Gangetic Delta [2]. During a single year (2011–2012), nine states sent strains of *V*. *cholerae* to the National Institute of Cholera and Enteric Disease, Kolkata [3]. Clearly, control efforts are needed urgently to control this public health problem in the country.

Cholera can be prevented, but the most appropriate methods need to be defined and adapted to the resource poor settings that exist in India, and the efforts need to be focused on areas and populations at highest risk. When cholera outbreaks occur, cases tend to be clustered in certain areas and among certain population groups [4, 5], requiring a good understanding of spatial epidemiology of the disease. A study that describes the spatial distribution of disease, its incidence using Geographic Information Science (GIS) and its association to potential risk factors should help guide interventions to control cholera [6].

With support from the Bill and Melinda Gates Foundation, a Cholera Expert Group was formed in India with the intent of preparing a road map for prevention and control of cholera in India. Accordingly, they gather data from existing sources that report outbreak cases in the country in order for understanding the burden of cholera and identify the areas where interventions are essentially needed. This study, by using the data gathered by them, describes spatial distribution of cholera in India, identifies cholera burden hotspots and factors associated with the risk for cholera. A burden hotspot defines an area of elevated disease prevalence or incidence [7].

## Material and methods

### Cholera data

District level cholera case reports from 2010 to 2015 were obtained from Integrated Disease Surveillance Programme (IDSP) of National Centre for Disease Control (NCDC), Government of India (http://idsp.nic.in/). The IDSP was launched with World Bank assistance in November 2004. One of the most important objectives of IDSP is strengthening of the Disease Surveillance System for epidemic prone diseases to detect and respond to outbreaks. An outbreak is defined as the occurrence of a disease or syndrome clearly in excess (or more than expected) in a given area (such as clustering of cases), over a particular period of time or among a specific group of people. Note that we used the outbreak data on cholera in this study, because this is the only data on cholera in India are available throughout the country since IDSP launched this program. The IDSP receives information regarding disease outbreaks (any unusual increase in cases/deaths in an area) reports from all the 36 states/union territories weekly through its IDSP portal. This information on disease outbreaks are compiled and reported via the Weekly Outbreak Report which is available on the Website. Currently, about 90% of the districts are reporting weekly disease surveillance data under IDSP. Overall the percentage of districts providing timely surveillance reports consistently has improved since 2014.

The IDSP uses the WHO case definition [8], a case of cholera should be suspected when (i) in an area where the disease is not known to be present, a patient aged 5 years or more develops severe dehydration or dies from acute watery diarrhea; (ii) in an area where there is a cholera epidemic, a patient aged 5 years or more develops acute watery diarrhea, with or without vomiting. If cholera cases were detected in an area during 3 of out of 5 consecutive years, then the area is defined as endemic. This definition is in line with the WHO Strategic Advisory Group of Experts on Immunization [9]. The “area” may be a region, district or an entire country, depending on the epidemiological setting.

### Population and socioeconomic data

Census 2011 population and other socio-economic data of India by district were obtained from the website of the Ministry of Home Affairs, Government of India (http://www.censusindia.gov.in/2011census/Hlo-series/hlo.html). Details on the census can be found in that website. In short, the census is the largest source of official statistical information on characteristics of the people of India, including reports on many variables related to health and health risks. The Office of the Registrar General and Census Commissioner, India under Ministry of Home Affairs, Government of India, is responsible for the census which has been conducted every ten years since 1872.

In the data source, “urban” is defined as a statutory town, census town, and outgrowth. All places with a municipality, corporation, cantonment board or notified town area committee, etc. are known as a “statutory town.” Places that satisfy the criteria of a minimum population of 5,000 with at least 75% of the male workers engaged in non-agricultural pursuits, and a density of population of at least 400/km^2^ are classified as a “census town.” An “outgrowth” is a village or part of village contiguous to a statutory town and possesses the urban features in terms of infrastructure and amenities such as hard surface roads, electricity, drainage system, education institutions, post offices, medical facilities, banks, etc. All areas that are not categorized as “urban” are considered “rural”. A “literate person” was defined as a person aged seven years or above able to read and write with understanding.

### GIS data

The digital district map of India was obtained from GitHub (https://github.com/datameet/maps/tree/master/Districts), which is shared under Creative Commons Attribution 2.5 India license. The map was created based on census 2011. In this study, the map was projected in WGS 1984 UTM zone 43N for conducting spatial analysis. In the analysis, we excluded a part of Jammu & Kashmir where detailed district boundaries and related data were not available. We used ArcGIS 10.4.1 (ESRI Inc.) for mapping the cholera risk in the country.

### Spatial clustering

For cluster detection, we used Poisson-based spatial scan statistic [10], implemented in SaTScan version 9.4.4 (http://www.satscan.org/). In this model, the number cholera cases in the districts were assumed Poisson distributed under the null hypothesis, and the underlying distribution of the population was adjusted for detection of the clusters. The centroids of the districts were used as the geographic references of the districts. Under the Poisson model, the expected number of cases in each part of the study area is proportional to its population size. The model detected “clusters” in a multidimensional point process and allowed variable window sizes to scan for cholera cases within the study area. We used variable window size, because we did not have prior knowledge about the size of the area covered by a “cluster”. We used a circular scan window, which moved over the entire study area. The radius of the window varied from zero to 20% of the population at risk. “Clusters” indicated areas with lower rates outside a circular scan window compared with higher rates inside a circular scan window. The location and size of the window changed, creating several distinct geographical circles. Since we used variable window sizes, computing the number of points at any given time was not possible [11]; therefore, a likelihood ratio was calculated. Under the Poisson model, the likelihood function for a specific window is:

$$\lambda ={\left(\frac{n}{\mu }\right)}^{n}{\left(\frac{N-n}{N-\mu }\right)}^{N-n}I\;(n>\mu )$$

where,

*N* = number of cases in the study area

*n* = number of cases within the window

*μ* = expected number of cases within the window under the null hypothesis

*I()* = an indicator function

Since we scanned for clusters with only the high rates, *I()* was 1 when the window had more cases than expected under the null hypothesis and in all other cases it was 0. The likelihood function was maximized over all windows, identifying the window that constituted the most likely cluster. The most likely cluster (hotspots) was the area that was least likely to have occurred by chance. The likelihood ratio for the window was noted and constituted the maximum likelihood ratio test statistic. Its distribution under the null hypothesis and its corresponding p-value were determined by repeating the same procedure on a large number of random replications of the data set generated under the null hypothesis using a Monte Carlo simulation approach.

### Statistical analysis to identify the risk factors

To evaluate whether certain factors, such as water and sanitation, and socioeconomic variables were associated with risk of cholera, we used the zero-inflated Poisson (ZIP) model. The independent variables were the district level characteristics and the number of cholera cases in the district was the dependent variable. The log of the number of population in the district was used as offset in the model. The ZIP model was chosen because our data were a two-component mixture composed of at-risk districts whose responses (i.e. cholera cases) follow Poisson process and non-risk districts whose responses are constant, i.e., zero [12]. Statistically significant spatial clustering of residuals (model over- and under-predictions) is evidence that your model is missing key explanatory variables. One can use the spatial autocorrelation tool to determine whether the spatial clustering of regression model residuals is statistically significant or not. We tested spatial autocorrelation of the model residuals using Global Moran’s I implemented in GeoDa version 1.8.12 to evaluate whether any important spatially related independent variable was missing in the regression model. Using the Poisson model, we then predicted cholera cases for each district based on the factors identified as being significant (p<0.05). SAS version 9.3 was used to analyze the risk factor analysis and for predicting cholera.

### Ethics

The study used secondary data aggregated at the district level, thus it did not require ethical approval.

### Role of funding agencies

The sponsor of the study had no role in study design, data collection, analysis, or interpretation, or writing of the report. The corresponding author had full access to all the data in the study and had final responsibility for the decision to submit for publication.

## Results

**Fig 1:**
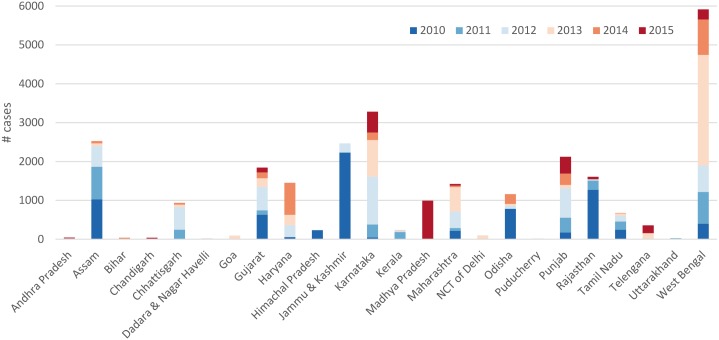
Number of observed cholera cases by States in India, 2010–2015.

There were 27,615 reported cholera cases during 2010–2015 with the highest number of cases (7,330) in 2010 and lowest (2,702) in 2015. Of 24 of the 36 states (67%) and 150 of 641 (23%) districts reported cholera in at least one of the study years. These districts made up 31% of the total population of India. State wise cumulative number of cases over the 6-year period are presented in Table 1. A high number of cases were observed in Jammu & Kashmir in particular due to a large outbreak in 2010 associated with heavy rains as per “The Hindu” (http://www.thehindu.com/news/national/other-states/Diarrhoea-cholera-outbreak-in-seven-districts-of-JampK/article15768616.ece). However, the highest burden of cholera was in West Bengal with 5,914 cases in the 6 years (Fig 1). In 13 states (Assam, Chandigarh, Chhattisgarh, Gujarat, Haryana, Karnataka, Kerala, Maharashtra, Odisha, Punjab, Rajasthan, Tamil Nadu, and West Bengal) cholera was reported during at least three out of five consecutive years, and were defined as cholera endemic states. Five of these states: Gujarat, Karnataka, Maharashtra, Punjab and West Bengal reported cholera in each of the years studied.

**Table 1:** Cumulative five-year (2010–2015) total cholera cases due to outbreaks by States in India.

State	Total no. of districts	Area (km^2^)	Population	No. of districts with cholera	No. years affected	% of population at risk	Total no. of cases
Andaman & Nicobar Island	3	8,252	379,944	0	0	0.00	0
Andhra Pradesh	13	161,290	49,378,776	2	2	16.46	45
Arunanchal Pradesh	16	90,090	1,382,611	0	0	0.00	0
Assam	27	84,880	31,169,272	12	5	47.33	2,526
Bihar	38	96,745	103,804,637	2	1	7.60	41
Chandigarh	1	118	1,054,686	1	3	100.00	39
Chhattisgarh	18	136,936	2,554,0196	6	4	33.09	937
Dadara & Nagar Havelli	1	494	342,853	1	2	100.00	15
Daman & Diu	2	109	242,911	0	0	0.00	0
Goa	2	3,627	1,457,723	1	1	56.10	97
Gujarat	26	186,302	60,383,628	12	6	53.34	1,842
Haryana	21	44,108	25,353,081	8	5	36.68	1,451
Himachal Pradesh	12	55,726	6,856,509	1	1	11.86	235
Jammu & Kashmir	23	222,363	12,548,926	2	2	13.96	2,467
Jharkhand	24	82,172	32,966,238	0	0	0.00	0
Karnataka	30	191,707	61,130,704	23	6	84.00	3,284
Kerala	14	38,809	33,387,677	3	4	14.18	237
Lakshadweep	1	664	64,429	0	0	0.00	0
Madhya Pradesh	50	309,061	72,597,565	4	3	10.49	995
Maharashtra	35	307,880	112,372,972	14	6	46.74	1,422
Manipur	9	24,412	2,721,756	0	0	0.00	0
Meghalaya	7	23,976	2,964,007	0	0	0.00	0
Mizoram	8	22,908	1,091,014	0	0	0.00	0
NCT of Delhi	9	1,504	16,753,235	2	1	14.48	100
Nagaland	11	18,229	1,980,602	0	0	0.00	0
Odisha	30	159,600	41,947,358	7	4	31.09	1,160
Puducherry	4	504	1,244,464	1	2	76.06	17
Punjab	20	50,393	27,704,236	9	6	63.41	2,122
Rajasthan	33	342,598	68,621,012	7	4	28.56	1,607
Sikkim	4	7449	607,688	0	0	0.00	0
Tamil Nadu	32	130,238	72,138,958	16	5	60.15	677
Telengana	10	115,159	35,286,757	2	2	20.05	358
Tripura	4	11,217	3671032	0	0	0.00	0
Uttar Pradesh	71	242,646	199,581,477	0	0	0.00	0
Uttarakhand	13	53,877	10,116,752	1	1	19.05	27
West Bengal	19	89,034	91,347,736	13	6	69.80	5,914
**Total**	**641**	**3,315,077**	**1,210,193,422**	**150**		**30.69**	**27,615**

The basic statistics of the study variables by group are shown in Table 2. Initially, we created bivariate models taking account of each variable in the model along with the number of cholera cases as the outcome. This analysis found that several variables were associated with risk for cholera in the district (S1 Table). Since multicollinearity is a problem for the multivariable model, we selected only one variable from each group for the final model to avoid collinearity in the data (S2 Table). In selecting the variables from a group, we also checked collinearity of the variables across the groups. For instance, we observed a high collinearity between percentage of urban area and percentage of households owning a television (TV) in the district. Therefore, we selected “owning of mobile phone” instead of “owning TV” as the proxy for the socioeconomic status, which did not show collinearity with the urban residency.

**Table 2:** Socioeconomic status of the districts in India, 2011.

Variable	Mean	Median	Std Dev	Minimum	Maximum
**Socioeconomic**					
% literate in the district	72.25	72.15	10.64	27.73	97.91
% literate male in the district	80.30	81.42	9.11	28.65	98.63
% literate female in the district	63.70	63.09	12.92	26.88	97.67
% of urban area in the district	9.23	2.51	21.46	0.00	100.00
% households using electricity in the district	65.75	76.02	28.42	0.06	99.81
Population density (km^2^) in the district	990.55	367.61	3729.67	0.97	46884.11
% households owning television in the district	43.53	40.26	24.15	0.00	95.40
% households owning computer in the district	8.01	6.59	5.19	0.00	39.32
% households owning mobile telephone in the district	50.88	53.23	15.05	0.00	79.62
**Water sources**					
% households using tap water from treated source in the district	28.24	21.66	23.45	0.85	99.60
% households using tap water from untreated source in the district	13.72	8.18	14.72	0.28	73.39
% households using water from covered well in the district	1.76	0.85	3.06	0.01	33.03
% households using water from uncovered well in the district	10.78	5.53	13.84	0.01	73.11
% households using hand pump in the district	31.51	22.42	29.20	0.00	97.15
% households using tubewell/borehole in the district	7.39	4.58	7.77	0.00	40.91
% households using spring in the district	1.75	0.10	4.93	0.00	45.70
% households using river/canal in the district	1.54	0.31	3.64	0.00	32.59
% households using tank/pond in the district	1.54	0.26	4.96	0.00	48.87
% households using other sources in the district	1.72	1.05	2.23	0.02	25.62
**Sanitation system**					
% households using piped sewer system in the district	8.88	3.80	13.79	0.18	92.72
% households using septic tank in the district	22.31	18.10	14.70	0.60	94.61
% households using other system in the district	3.10	1.58	4.23	0.05	52.77
% households using slab/ventilated improved pit in the district	7.25	3.75	8.93	0.08	67.95
% households using without slab/open pit in the district	3.50	0.57	7.41	0.01	38.56
% households disposing night soil into open drain in the district	0.54	0.25	1.24	0.00	20.18
% households removing night soil by human in the district	0.57	0.04	3.14	0.00	41.83
% households servicing night soil by animals in the district	0.34	0.15	1.19	0.00	18.34
% households using public latrine in the district	2.54	1.37	3.75	0.05	42.92
% households using open latrine in the district	50.94	54.63	27.30	0.00	93.33
**Drainage system**					
% households using closed drainage in the district	13.48	8.41	15.57	0.00	96.49
% households using open drainage in the district	33.73	30.81	19.99	0.00	88.50
% households had no drainage in the district	52.29	55.90	25.27	0.00	95.68

The results of the Poisson component of the multivariable model show that districts with higher literacy rates had a lower risk for cholera (rate ratio = 0.9717, 95% CI: 0.9703–0.9732, p<0.0001) (Table 3). This means if the literacy rate in a district is increased by 1 percentage point, the expected number of cases in the district will be decreased by 3% while holding all other variables in the model constant. We also observed similar results for literacy from the zero inflated component of the model (relative risk = 0.9730, 95% CI = 0.9526–0.9939, p = 0.0117). This indicates if the literacy rate in a district is increased by 1 percentage point, the risk of having cholera in the district will be decreased by 3%. The results of the zero inflated component of the model were very similar to the Poisson component except that “% households owning mobile telephone in the district” and “% households using latrine without slab/open pit in the district” did not yield to statistical significance. The results of the analysis of the spatial pattern of the regression residuals show no spatial patterns of the residuals (Moran’s I = -0.0050, p = 0.47).

**Table 3:** Associations between district level characteristics and the number of cholera in the district in a multivariable model.

Variables	Rate ratio/ Relative risk*	95% CI	Chi-square	P-value
**Poisson component (count model)**				
% literate in the district	0.9717	0.9703–0.9732	1381.82	<0.0001
% of urban area in the district	1.0017	1.0002–1.0031	5.15	0.0233
% households owning mobile telephone in the district	0.9891	0.9880–0.9902	358.11	<0.0001
% households using tap water from treated source in the district	0.9911	0.9902–0.9919	436.43	<0.0001
% households using latrine without slab/open pit in the district	1.0207	1.0188–1.0226	477.61	<0.0001
% households using open drainage	1.0178	1.0170–1.0186	2197.82	<0.0001
**Zero inflated component (logistic model)**				
% literate in the district	0.9730	0.9526–0.9939	6.36	0.0117
% of urban area in the district	1.0228	1.0096–1.0359	11.72	0.0006
% households owning mobile telephone in the district	1.0126	0.9962–1.0291	2.26	0.1326
% households using tap water from treated source in the district	0.9755	0.9650–0.9861	20.19	<0.0001
% households using latrine without slab/open pit in the district	1.0194	0.9897–1.0499	1.62	0.2034
% households using open drainage	1.0172	1.0054–1.0291	8.28	0.0040

*The risk ratios are or the Poisson component of the model and the relative risks are for the zero inflated component of the model.

**Fig 2:**
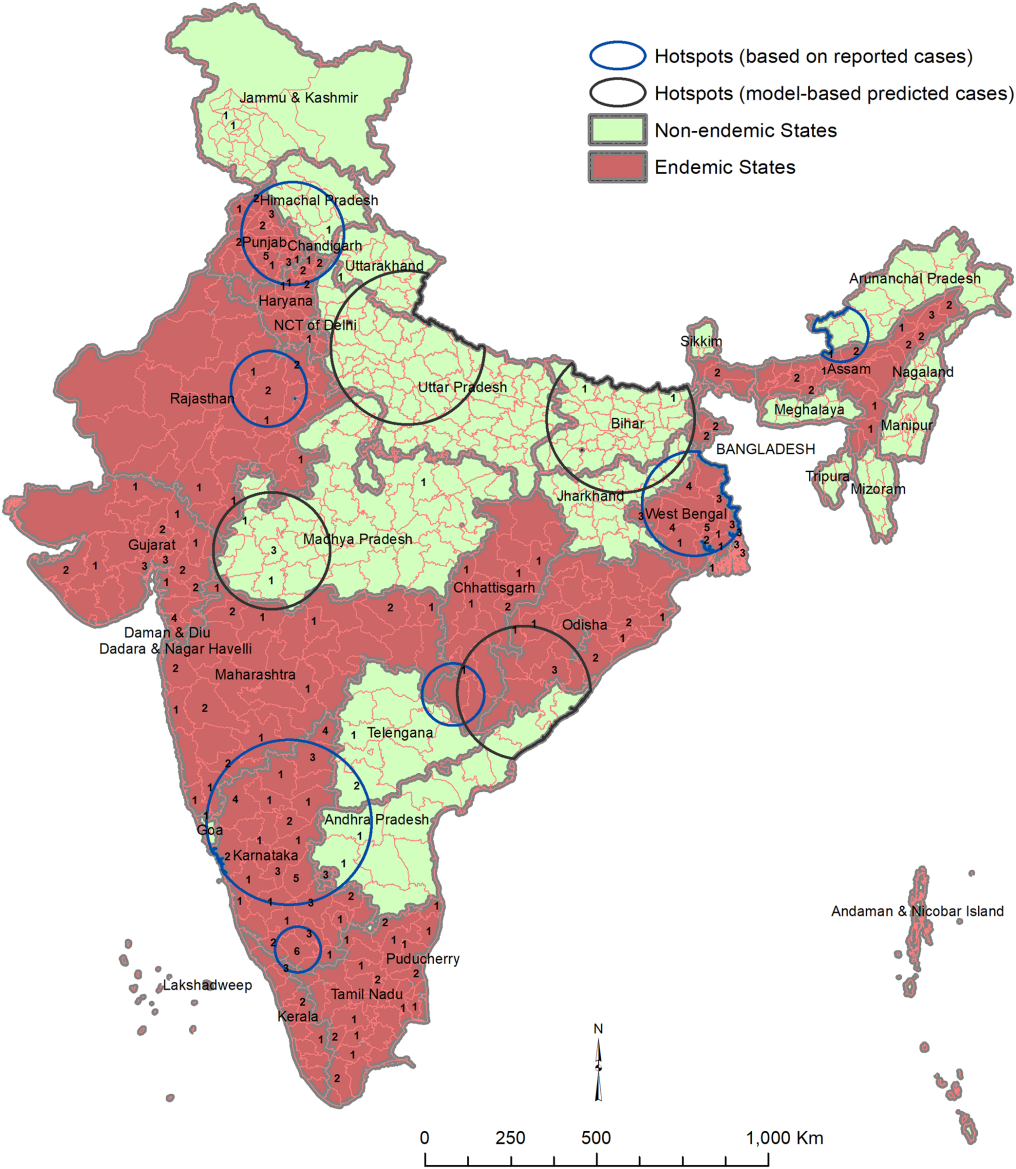
Spatial patterns of cholera and the high-risk areas (hot spots). Note: The numbers inside districts indicate no. of years of outbreak during 2010–2015.

The results of the SatScan for cluster detection yielded seven significantly high-risk clusters of different sizes from reported cholera cases and four high-risk clusters from the multivariable model-based predicted cholera cases (Fig 2). 78 districts in 15 States from reported cholera cases and 111 districts from predicted cholera cases were centered in these high-risk clusters (Table 4). The largest cluster detected from reported cholera cases was in Southern India primarily in districts of Karnataka. The second largest cluster was in West Bengal, and the third was in Northern part of India comprising of Punjab and adjoining areas of Himachal Pradesh. The high-risk clusters based on predicted cases were very large and mostly in Bihar, Madhya Pradesh, Uttar Pradesh and Odisha.

**Table 4:** High risk districts for cholera in India.

States	# districts	Districts
**Based on observed cholera cases**		
Andhra Pradesh	2	Anantapur, Kurnool
Arunanchal Pradesh	3	East Kameng, Tawang, West Kameng
Assam	2	Sonitpur, Udalguri
Chandigarh	1	Chandigarh
Chhattisgarh	2	Bijapur, Dakshin Bastar Dantewada
Goa	1	South Goa
Haryana	5	Ambala, Kaithal, Kurukshetra, Panchkula, Yamunanagar
Himachal Pradesh	9	Bilaspur, Hamirpur, Kangra, Kullu, Mandi, Shimla, Sirmaur, Solan, Una
Jharkhand	5	Deoghar, Dhanbad, Dumka, Jamtara, Pakur
Karnataka	19	Bagalkot, Belgaum, Bellary, Bijapur, Chikmagalur, Chitradurga, Davanagere, Dharwad, Gadag, Gulbarga, Haveri, Koppal, Mandya, Mysore, Raichur, Shimoga, Tumkur, Uttara Kannada, Yadgir
Maharashtra	1	Sangli
Punjab	13	Barnala, Fatehgarh Sahib, Gurdaspur, Hoshiarpur, Jalandhar, Kapurthala, Ludhiana, Moga, Patiala, Rupnagar, Sahibzada Ajit Singh Nagar, Sangrur, Shahid Bhagat Singh Nagar
Rajasthan	4	Jaipur, Sikar, Tonk, Dausa
Telengana	1	Mahbubnagar
West Bengal	10	Bankura, Barddhaman, Birbhum, Haora, Hugli, Kolkata, Murshidabad, Nadia, Pashchim Medinipur, Puruliya
**Based on predicted cholera cases**		
Andhra Pradesh	3	Srikakulam, Visakhapatnam, Vizianagaram
Bihar	37	Araria, Aurangabad, Banka, Begusarai, Bhagalpur, Bhojpur, Buxar, Darbhanga, Gaya, Gopalganj, Jamui, Katihar, Khagaria, Kishanganj, Lakhisarai, Madhepura, Madhubani, Munger, Muzaffarpur, Nalanda, Nawada, Pashchim Champaran, Patna, Purba Champaran, Purnia, Rohtas, Saharsa, Samastipur, Saran (chhapra), Sheikhpura, Sheohar, Sitamarhi, Siwan, Supaul, Vaishali, Arwal, Jehanabad
Chhattisgarh	3	Bastar, Dakshin Bastar Dantewada, Narayanpur
Jharkhand	7	Chatra, Deoghar, Giridih, Godda, Hazaribagh, Kodarma, Sahibganj
Madhya Pradesh	16	Alirajpur, Barwani, Bhind, Burhanpur, Dewas, Dhar, East Nimar, Harda, Indore, Jhabua, Rajgarh, Ratlam, Sehore, Shajapur, Ujjain, West Nimar
Odisha	7	Gajapati, Kalahandi, Koraput, Malkangiri, Nabarangapur, Nuapada, Rayagada
Rajasthan	1	Banswara
Uttar Pradesh	33	Agra, Aligarh, Auraiya, Bahraich, Ballia, Bara Banki, Bareilly, Bijnor, Budaun, Bulandshahr, Etah, Etawah, Farrukhabad, Firozabad, Hardoi, Mahamaya Nagar, Jalaun, Jyotiba Phule Nagar, Kannauj, Kanpur Dehat, Kanpur Nagar, Kansiram Nagar, Kheri, Lucknow, Mainpuri, Mathura, Moradabad, Pilibhit, Rampur, Shahjahanpur, Shrawasti, Sitapur, Unnao
Uttarakhand	4	Almora, Champawat, Nainital, Udham Singh Nagar

## Discussion

Our study shows that one-fourth of the districts (150/641) reported cholera and 90 districts were identified as hotspots, which are most likely clusters as obtained from results of the analysis of spatial clustering. Thus, cholera is a wide-spread major public health problem in India, but particularly in the states of West Bengal, Karnataka, Punjab, and areas of Himachal Pradesh adjoining Punjab. The risk of cholera in West Bengal is not surprising, because historically Asiatic cholera has always thrived in the Ganges river delta region, the greater part of which is now Bangladesh [13, 14]. In fact, the majority of cases in India (49%) came from West Bengal which borders Bangladesh [15]. Note that only 7% of the total Indian population resides in West Bengal.

In addition to West Bengal, most of the districts in Karnataka were identified as cholera hotspots. This increased risk in Karnataka may be related to acute shortage of safe water and poor environmental sanitation during pre-monsoon and early monsoon seasons [16]. There are also several other pockets of hotspots in different regions, such as Rajasthan and Chhattisgarh, probably due to poor environmental hygiene, lack of access to potable water and risk-posing lifestyles (e.g. not washing hands properly before meal, eating street food, etc.). Several socioeconomic factors including water and sanitation conditions are associated with increased risk of the disease so it is not surprising that districts with poor water and sanitation indicators were at increased risk for cholera.

Our study also shows that districts with more urban areas had a higher risk. Cholera outbreaks have occurred in various urban areas [17–19]; however, we observed a higher number of cases coming from rural areas than urban areas during 2012–2015 (column “Comments” in S3 Table). One explanation for this finding could include more complete reporting from districts with more cities, even though the cases are occurring in the rural area. Alternatively, water sanitation conditions might be allocated preferentially to urban areas in the districts with more cities. To further explore this possibility, we reviewed UNICEF’s sanitation database (http://data.unicef.org/water-sanitation/sanitation.html) which reports a lower rate of improved sanitation in rural areas. Use of improved sanitation facilities in urban area in India had increased from 49% in 1990 to 63% in 2015, whereas it has increased from 6% in 1990 to 28% in 2015 in the rural areas, illustrating a wide gap in sanitation status between rural and urban areas in India. Over the last few decades India’s planning process has increasingly recognized the need to minimize the rural-urban gap, but still differences persist [20]. It appears that families living in rural areas of districts with more cities are at higher risk, perhaps because the resources in these districts are allocated to the urban areas, neglecting the rural areas of the district.

In our study, open drainage system also poses a higher risk for cholera. A good sewage facility is important for complete evacuation of waste materials. In an open drainage system, the fecal materials from latrines may overflow during the rainy season, thus discharging the contents directly into the environment [21], leading to increased risk of the disease. The higher risk was also associated with poverty. The proxy that we used for higher socioeconomic status of the household (owning mobile phone) reiterates that cholera is a disease of poverty [22–24].

The primary strength of our study is the access to district level data on reported cases of cholera and the ability to relate this to the socioeconomic data from Census 2011 from the Ministry of Home Affairs, Government of India. This census data, which was systematically collected from all districts ensured greater reliability of the data. A limitation of our study is that the cholera data were not community-based, which precluded calculating the absolute risk of the disease. Conducting national level community-based disease surveillance over a long period is unrealistic. However, the data used in this study provided a basis for understanding relative burden of cholera across the regions. Additionally, since the data came from the national surveillance conducted by the IDSP and was systematically executed throughout India, we believe the burden of cholera in the different districts are comparable.

One of the limitations in our study is that many of the cholera cases were not culture confirmed, although outbreaks are frequently confirmed by culturing a sample of specimens early in an outbreak. Another potential weakness is that IDSP only reports positive cases from outbreaks and not from sporadic cases. Some states appear to have a better reporting system (e.g. Karnataka) and may report proportionately more cases than states with poor reporting systems. For example, Uttar Pradesh reported no cases despite having areas that qualify as high risk. This emphasizes the need to strengthen the current cholera surveillance to improve the information on cholera, especially in those states which are not currently reporting cases. Surveillance could be enhanced with the appropriate use of rapid diagnostic tests, especially at the district health care facilities and in tertiary care hospitals in urban areas. Another issue is that the hotspots were defined only in space using cumulative counts over the years. We observed only a few districts (4%) with cholera cases at least 3 of the 6 years, thus the data were not suitable for performing spatiotemporal clusters analysis or broken down by year.

In India, cholera cases are largely underreported for several reasons. These include limited disease surveillance, inadequate laboratory capacity especially at the peripheral health-care centers, and reluctance to acknowledge the problem by authorities for fear of societal repercussion [2]. To overcome the limitation of the data for identifying hotspots, we used predicted counts of cholera cases from the multivariable model based on the factors that explained the risk of the disease. This model suggests that cholera is occurring not only in places that report cases but is also highly likely to be occurring in several other places such as Bihar, Madhya Pradesh, Uttar Pradesh and Odisha. Since the residuals of the zero inflated model were not spatially heterogeneous as obtained from Moran’s I test, the results of the model were not affected by missing of an important spatially related variable for identifying risk factors. Another limitation is that we used population data of only one year (census 2011) as a representative population size of the districts. However, we do not think that would affect in identification of hotspots, because relative difference of the population size across the districts would not change had we averaged the population size of the different years.

In a study conducted in Philippines [25], district level coverage of improved water sources was found positively associated and improved sanitation system was negatively associated with the risk for cholera when using the data of both confirmed and suspected cases. When only the confirmed cases were analyzed in that study, only the sanitation coverage was found to be negatively associated with the risk for cholera. The investigators of that study believed that the positive association of cholera with improved water source was due to breakdown in the infrastructure and non-chlorination of water supplies illustrating that proper management of the infrastructure is important for ensuring improved source of water. In our study, an increased risk for cholera is largely associated with the coverages of both water and sanitation conditions in the district. Therefore, controlling the disease may be a challenge without major improvements in providing safe water and sanitation [26, 27]. Improving and proper management of this infrastructure is made more complex by the continued population growth and the human migration within India, and these changes could even alter the locations of hotspots in the future. Very large investments in water and sanitation would be needed to significantly reduce cholera risk [28]. Even if achieved, improving water quality is complicated since water may become contaminated during collection or when stored and used within the household as a result of poor hygiene [29, 30]. Treating water with chlorine at the household level for prolonged periods has generally not been acceptable to the population [28], and expecting sustainable behavior change by simply distributing chlorine or other forms of household water treatment has not been realistic.

An oral cholera vaccine (Shanchol) is being produced in India, and has been found to be safe and effective for at least 5 years [31, 32]. As an immediate solution, we believe rational use of the oral cholera vaccine (OCV) could be an effective control mechanism for cholera in India [33], but it will need to be integrated with long-term water and sanitation improvements. A mass vaccination campaign conducted in Odisha, India found that cholera vaccination was feasible using Indian government’s public health infrastructure [34].

The results of our study identified several hotspots of cholera where cholera interventions should be focused and policymakers could use this risk map when developing their national plan for controlling cholera in India.

## Supporting information

S1 Table Associations between district level characteristics and the number of cholera in the district in a bivariate model. (DOCX)

S2 Table Results of the multicollinearity test of the groups of variables. (DOCX)

S3 Table Cholera outbreaks details 2012–2015. (DOCX)
