# Supplementary material for: Identification of burden hotspots and risk factors for cholera in India: An observational study
Source: PLoS One. 2017 Aug 24;12(8):e0183100. doi: 10.1371/journal.pone.0183100 (PMC5570499; doi:10.1371/journal.pone.0183100)
Supplement: S1 Table — (DOCX) [file pone.0183100.s001.docx]

Table S1. Associations between district level characteristics and the number of cholera in the district in a bivariate model

| **Variables** | **Estimate** | **Standard**  **Error** | **95% CI** | **P-value** |
| --- | --- | --- | --- | --- |
| ***Socioeconomic*** | | | | |
| % literate in the district | -0.0409 | 0.0006 | -0.0421 to-0.0397 | <.0001 |
| % literate male in the district | -0.0491 | 0.0007 | -0.0505 to -0.0476 | <.0001 |
| % literate female in the district | -0.0324 | 0.0005 | -0.0334 to -0.0314 | <.0001 |
| % of urban area in the district | -0.0311 | 0.0009 | -0.0329 to -0.0293 | <.0001 |
| % households using electricity in the district | -0.0091 | 0.0002 | -0.0095 to -0.0086 | <.0001 |
| Population density (km^2^) in the district | -0.0001 | 0.0000 | -0.0001 to -0.0001 | <.0001 |
| % households owing television in the district | -0.0133 | 0.0003 | -0.0139 to -0.0128 | <.0001 |
| % households owning computer in the district | -0.0531 | 0.0012 | -0.0554 to -0.0508 | <.0001 |
| % households owning mobile telephone in the district | -0.0192 | 0.0004 | -0.0200 to -0.0183 | <.0001 |
| ***Water sources*** | | | | |
| % households using tap water from treated source in the district | -0.0157 | 0.0003 | -0.0163 to -0.0152 | <.0001 |
| % households using tap water from untreated source in the district | 0.0005 | 0.0005 | -0.0004 to 0.0014 | 0.3052 |
| % households using water from covered well in the district | -0.0138 | 0.0041 | -0.0219 to -0.0058 | 0.0007 |
| % households using water from uncovered well in the district | 0.0105 | 0.0005 | 0.0095 to 0.0115 | <.0001 |
| % households using hand pump in the district | 0.0058 | 0.0002 | 0.0053 to 0.0063 | <.0001 |
| % households using tubewell/borehole in the district | 0.0157 | 0.0007 | 0.0143 to 0.0171 | <.0001 |
| % households using spring in the district | 0.1264 | 0.0026 | 0.1213 to 0.1315 | <.0001 |
| % households using river/canal in the district | 0.1974 | 0.0012 | 0.1949 to 0.1998 | <.0001 |
| % households using tank/pond in the district | -0.0119 | 0.0040 | -0.0198 to -0.0041 | 0.0029 |
| % households using other sources in the district | -0.1192 | 0.0072 | -0.1332 to -0.1052 | <.0001 |
| ***Sanitation system*** | | | | |
| % households using piped sewer system in the district | -0.0142 | 0.0004 | -0.0150 to -0.0135 | <.0001 |
| % households using septic tank in the district | -0.0095 | 0.0006 | -0.0106 to -0.0084 | <.0001 |
| % households using other system in the district | 0.1448 | 0.0019 | 0.1410 to 0.1485 | <.0001 |
| % households using slab/ventilated improved pit in the district | -0.0038 | 0.0006 | -0.0049 to -0.0026 | <.0001 |
| % households using without slab/open pit in the district | 0.0307 | 0.0008 | 0.0290 to 0.0323 | <.0001 |
| % households disposing night soil into open drain in the district | 0.0211 | 0.0105 | 0.0005 to 0.0417 | 0.0444 |
| % households removing night soil by human in the district | 0.0874 | 0.0005 | 0.0863 to 0.0884 | <.0001 |
| % households servicing night soil by animals in the district | 1.1891 | 0.0266 | 1.1370 to 1.2411 | <.0001 |
| % households using public latrine in the district | -0.0743 | 0.0017 | -0.0776 to -0.0709 | <.0001 |
| % households using open latrine in the district | 0.0012 | 0.0002 | 0.0007 to 0.0017 | <.0001 |
| ***Drainage system*** | | | | |
| % households using closed drainage in the district | -0.0190 | 0.0004 | -0.0197 to -0.0183 | <.0001 |
| % households using open drainage in the district | 0.0109 | 0.0003 | 0.0103 to 0.0116 | <.0001 |
| % households had no drainage in the district | 0.0044 | 0.0002 | 0.0040 to 0.0049 | <.0001 |
