# Supplementary material for: Identification of burden hotspots and risk factors for cholera in India: An observational study
Source: PLoS One. 2017 Aug 24;12(8):e0183100. doi: 10.1371/journal.pone.0183100 (PMC5570499; doi:10.1371/journal.pone.0183100)
Supplement: S2 Table — (DOCX) [file pone.0183100.s002.docx]

Table S2. Results of the multicollinearity test of the groups of variables

| Variable | Parameter  estimate | Standard Error | t-Value | P-value | Variance Inflation factor (VIF) |
| --- | --- | --- | --- | --- | --- |
| ***Educational status*** | | | | | |
| % literate in the district | -13.7020 | 20.45 | -0.67 | 0.5032 | 1112.95 |
| % literate male in the district | 5.6003 | 10.66 | 0.52 | 0.5998 | 221.93 |
| % literate female in the district | 7.8166 | 9.93 | 0.79 | 0.4319 | 387.61 |
| ***Urbanization and population density*** | | | | | |
| % of urban area in the district | -0.3793 | 0.35 | -1.06 | 0.2902 | 1.39 |
| Population density (km^2^) in the district | 0.0004 | 0.002 | 0.20 | 0.8410 | 1.39 |
| ***Economic status*** | | | | | |
| % households using electricity in the district | 0.4936 | 0.42 | 1.15 | 0.2506 | 3.50 |
| % households owing television in the district | -0.0545 | 0.62 | -0.09 | 0.9302 | 5.32 |
| % households owning computer in the district | 0.9059 | 1.62 | 0.56 | 0.5766 | 1.67 |
| % households owning mobile telephone in the district | -0.4601 | 0.52 | -0.88 | 0.3802 | 1.46 |
| ***Water sources*** |  |  |  |  |  |
| % households using tap water from treated source in the district | 77.1591 | 43.18 | 1.79 | 0.0744 | 25628.00 |
| % households using tap water from untreated source in the district | 77.0584 | 43.19 | 1.78 | 0.0749 | 10106.00 |
| % households using water from covered well in the district | 75.8202 | 43.20 | 1.75 | 0.0798 | 437.61.00 |
| % households using water from uncovered well in the district | 76.7494 | 43.17 | 1.78 | 0.0760 | 8926.88 |
| % households using hand pump in the district | 76.6789 | 43.16 | 1.78 | 0.0762 | 39689.00 |
| % households using tubewell/borehole in the district | 81.3386 | 43.15 | 1.88 | 0.0599 | 2815.46 |
| % households using spring in the district | 75.4194 | 43.19 | 1.75 | 0.0813 | 1134.59 |
| % households using river/canal in the district | 82.9877 | 43.22 | 1.92 | 0.0553 | 618.41 |
| % households using tank/pond in the district | 75.0664 | 43.19 | 1.74 | 0.0827 | 1148.65 |
| % households using other sources in the district | 70.5437 | 43.19 | 1.63 | 0.1030 | 232.46 |
| ***Sanitation system*** |  |  |  |  |  |
| % households using piped sewer system in the district | -1.4861 | 1.58 | -0.94 | 0.3484 | 12.18 |
| % households using septic tank in the district | -2.5997 | 1.64 | -1.58 | 0.1140 | 14.91 |
| % households using other system in the district | -6.1072 | 2.71 | -2.25 | 0.0249 | 3.38 |
| % households using slab/ventilated improved pit in the district | -0.3725 | 1.76 | -0.21 | 0.8333 | 6.38 |
| % households using without slab/open pit in the district | -2.1723 | 1.97 | -1.10 | 0.2721 | 5.48 |
| % households disposing night soil into open drain in the district | -7.0906 | 5.50 | -1.29 | 0.1984 | 1.195 |
| % households removing night soil by human in the district | 13.5057 | 2.68 | 5.03 | <.0001 | 1.82 |
| % households servicing night soil by animals in the district | -2.9808 | 5.60 | -0.53 | 0.5949 | 1.13 |
| % households using public latrine in the district | -1.6207 | 2.33 | -0.69 | 0.4880 | 1.96 |
| % households using open latrine in the district | -2.2628 | 1.57 | -1.43 | 0.1522 | 47.43 |
| ***Drainage system*** |  |  |  |  |  |
| % households using closed drainage in the district | -1.1855 | 1.22 | -0.97 | 0.3337 | 8.57 |
| % households using open drainage in the district | -1.7149 | 1.17 | -1.46 | 0.1453 | 13.02 |
| % households had no drainage in the district | -1.5743 | 1.15 | -1.36 | 0.1729 | 20.01 |
