# Supplementary material for: Identification of burden hotspots and risk factors for cholera in India: An observational study
Source: PLoS One. 2017 Aug 24;12(8):e0183100. doi: 10.1371/journal.pone.0183100 (PMC5570499; doi:10.1371/journal.pone.0183100)
Supplement: S3 Table — (DOCX) [file pone.0183100.s003.docx]

| **Table S3. Cholera Outbreaks Details 2012-2015** | | | |  |  |  |  |  |  |  |
| --- | --- | --- | --- | --- | --- | --- | --- | --- | --- | --- |
| Wk No. | Year | State | District | Disease | Cases | Deaths | Date of Outbreak | Date of Reporting | Current Status | Comments |
| 1 | 2012 | Tamil Nadu | Theni | Cholera | 21 | 0 | 1/3/2012 | 1/7/2012 | Under control | Outbreak reported from Village Patchaiappapuram, PHC Varusanadu, and Block K.Mayiladumparai due to contamination of water. District RRT investigated the outbreak. 3 Stools collected out of which one positive for cholera culture. House to house survey done. Chlorination and disinfection of water sources done. Health education regarding proper water handling done. |
| 1 | 2012 | Tamil Nadu | Cuddalore | Cholera | 17 | 0 | 1/1/2012 | 1/7/2012 | Under control | Cases reported from Village/ PHC Srinedunseri, Block Kattumannar koil. Due to consumption of contaminated water. Block medical team investigated the outbreak. 5 stool samples collected, all found positive for Cholera culture. Chlorination and disinfection of water sources done. |
| 3 | 2012 | Tamil Nadu | Dindigul | Cholera | 16 | 0 | 1/15/2012 | 1/22/2012 | Under control | Cases reported from Village Nilakottai, PHC S.Thummalapatty due to consumption of contaminated water caused by leakage in water pipeline. District RRT visited the affected area and investigated the outbreak. Out of 8 stool samples collected and sent to MMC, Madurai, 2 samples tested positive for cholera culture. 4 cases admitted in IDH Austinpatti. House to house survey done. Instruction given to local authorities to ensure proper chlorination and repair of pipeline leakages. ORS packets and chlorine tablets distributed in the locality. IEC activities undertaken regarding safe drinking water, hygienic practices and sanitation measures. |
| 4 | 2012 | Karnataka | Belgaum | Cholera | 24 | 0 | 1/26/2012 | 1/28/2012 | Under control | Outbreak reported from Village Achamatti, Kagadal, PHC Haralikatti, Taluk Savadatti probably due to consumption of contaminated water caused by leakage in water pipeline and damaged overhead tank. District RRT visited the affected area and investigated the outbreak. Out of 2 stool samples collected, one tested positive for V. cholerae culture at Belgaum Medical Sciences Reference Lab. Emergency medical care started in the affected village. Alternative safe drinking water made available. Halogen tablets and ORS supplied house to house. Leakage in water pipeline repaired. Health education given regarding hygiene and sanitation. |
| 4 | 2012 | Tamil Nadu | Dindigul | Cholera | 10 | 0 | 1/19/2012 | 1/23/2012 | Under surveillance | Cases reported from Village Kalkottai, HSC Sirunaickenpatti, PHC Sirunaickenpatti, Block Nilakkottai due to leakage of water pipeline. District RRT visited the affected area and investigated the outbreak. Out of 2 stool samples collected and sent to MMC, Madurai, 1 found positive for Vibrio cholera by culture. House to house survey done for new cases. IEC activities undertaken regarding safe drinking water, hygienic practices and sanitation measures. |
| 5 | 2012 | Kerala | Idukki | Cholera | 4 | 0 | 1/24/2012 | 1/28/2012 | Under surveillance | Cases reported from Kattappana, PHC Pezhumkandam, Block Kanchiyar due to consumption of contaminated water (from mountain streams). District RRT visited the affected area and investigated the outbreak. Stool samples collected and sent for lab analysis to Microbiology lab, Kottayam; samples tested positive for hanging drop. One sample sent for stool culture tested positive for V. cholerae (Ogawa). House to house visit done by medical team. Water sources chlorinated. Emergency medical camp conducted to treat cases. IEC activities undertaken regarding safe drinking water, hygienic practices and sanitation measures. |
| 6 | 2012 | Karnataka | Chamarajnagar | Cholera | 72 | 0 | 1/28/2012 | 2/3/2012 | Under control | Outbreak reported from Village/PHC Kerehalli, Block Panyadhahundi. District RRT investigated the outbreak. 3 Stool samples collected out of which 1 was positive for cholera culture. ORS packets and chlorine tablets distributed in the locality. Health education given regarding hygiene and sanitation. |
| 9 | 2012 | Tamil Nadu | Madurai | Cholera | 12 | 0 | 2/26/2012 | 3/2/2012 | Under control | Cases reported from Village Karadikal, SC Karadikal, PHC Checkanurani, Block Thirumangalam of District Madurai. District RRT investigated the outbreak. Stool samples collected and sent to Madurai Medical College which were tested positive for Cholera by culture. Chlorination of water sources done Pipeline leakages repaired. IEC activities undertaken regarding safe drinking water, hygienic practices and sanitation measures. |
| 11 | 2012 | Karnataka | Mysore | Cholera | 12 | 0 | 3/13/2012 | 3/14/2012 | Under control | Cases reported from Village Kalkunike, PHC Hunsur, District Mysore. District RRT investigated the outbreak. House to house survey done for finding new cases. Symptomatic treatment done. Out of 13 stool samples collected, 7 were positive for Cholera culture. ORS packets distributed in the affected area. IEC activities undertaken regarding safe drinking water, hygienic practices and sanitation measures. |
| 13 | 2012 | Tamil Nadu | Thanjavur | Cholera | 3 | 0 | 3/26/2012 | 4/1/2012 | Under Surveillance | Cases reported from Village Pancharam, HSC Vayalur, PHC Nagathi, Block Thanjavur. District RRT visited the area and investigated the outbreak. 2 water samples taken and sent to Thanjavur Medical College. 1 sample tested positive for Cholera by culture. Chlorination of water sources done and disinfection of affected houses done. Treatment to contacts given. Health education given. |
| 14 | 2012 | Karnataka | Mysore | Cholera | 181 | 0 | 4/4/2012 | 4/4/2012 | Under control | Outbreak reported from Villages Srirampura, Sharkarapura, PHC Nanjangud, Block Nanjangud, District Mysore. District RRT investigated the outbreak. House to house survey done for finding new cases. Symptomatic treatment done. Out of 10 stool samples collected, 2 were positive for Cholera culture. ORS packets distributed in the affected area. IEC activities undertaken regarding safe drinking water, hygienic practices and sanitation measures. |
| 14 | 2012 | Karnataka | Tumkur | Cholera | 27 | 1 | 4/6/2012 | 4/7/2012 | Under control | Outbreak reported from Village Honnavali, PHC Honnavali, Block Tiptur, District Tumkur. District RRT investigated the outbreak. House to house survey done for finding new cases. Out of 3 stool samples collected, 2 were positive for Cholera culture (serotype Ogawa). ORS packets distributed in the affected area. IEC activities undertaken regarding safe drinking water, hygienic practices and sanitation measures. |
| 18 | 2012 | Tamil Nadu | Dindigul | Cholera | 12 | 0 | 4/27/2012 | 4/30/2012 | Under control | Cases reported from Village Mottiyagoundanpatti, SC K. Ayyapatti, PHC Gopalpatti, Block Shanarpatti, District Dindigul. District RRT investigated the outbreak. 2 stool samples sent to Madurai Medical College. 1 sample tested positive for V.cholerae by culture. House to house survey conducted with treatment of cases and contacts, along with chlorination of water sources and education regarding water & food hygiene. Local government bodies are undertaking repair of water pipe lines etc. |
| 18 | 2012 | Karnataka | Tumkur | Cholera | 57 | 0 | 4/24/2012 |  | Under control | Outbreak reported from Village Basavanahalli, SC Reddihalli. PHC Holavanahalli, Block Koratagere, District Tumkur. District RRT investigated the outbreak. Out of 5 Stool samples collected one was positive for V.Cholerae (Ogawa). Chlorination of water sources done in the affected area. Health education given regarding hygiene and sanitation. |
| 19 | 2012 | Karnataka | Tumkur | Cholera | 45 | 0 | 5/4/2012 | 5/9/2012 | Under control | Outbreak reported from Village Rudrapura, PHC Baluvaneralu, Block Tiptur, District Tumkur. District RRT investigated the outbreak. 2 Stool samples collected and tested positive for V.cholerae (Ogawa strain). Disinfection of the surrounding areas done. Chlorination of water sources done. Health education given. |
| 19 | 2012 | Karnataka | Mysore | Cholera | 36 | 0 | 5/6/2012 | 5/7/2012 | Under control | Outbreak reported from Village Ibjala, PHC Chandravadi, Block Nanjangud, District Mysore. District RRT investigated the outbreak. 3 stool samples tested positive for Cholera by culture. House to house survey done. Temporary clinic organized in the affected area. IEC activities done. |
| 19 | 2012 | Karnataka | Mysore | Cholera | 44 | 0 | 5/8/2012 | 5/9/2012 | Under control | Outbreak reported from Village Vaddarahalli, PHC Kitturu, Block Periyapatna, District Mysore. Block Health team investigated the outbreak. 5 stool samples collected and tested positive for Cholera by culture. House to house surveillance done. Water pipe leakage repaired. IEC activities done. |
| 19 | 2012 | Kerala | Wayanad | Cholera | 13 | 0 | 5/5/2012 | 5/7/2012 | Under control | Cases reported from Village Noolpuzha, PHC Noolpuzha, Block Bathery, District Wayanad. District RRT investigated the outbreak. House to house survey done. Out of 4 stool samples collected, 1 sample tested positive for V. Cholerae (O1 Ogawa strain) by culture. Medical camps organized in the affected area. Chlorination of water sources done. ORS packets distributed. IEC activities done. |
| 19 | 2012 | Kerala | Wayanad | Cholera | 8 | 1 | 5/3/2012 | 5/7/2012 | Under control | Cases reported from Village Nadavayal, CHC Panamaram, Block Mananthavady, District Wayanad. District RRT investigated the outbreak. House to house survey done. Out of 2 stool samples collected, 1 tested positive for V. Cholerae (O1 Ogawa) by culture. Medical camps organized in the affected area. Chlorination of water sources done. ORS packets distributed in the affected area. IEC activities done. |
| 19 | 2012 | Tamil Nadu | Tiruchirapalli | Cholera | 14 | 1 | 5/5/2012 | 5/8/2012 | Under control | Cases reported from Village Sengudi, SC Alagiyamanavalam, PHC Sirugambur, Block Manachanallur, District Tiruchirapalli possibly due to consumption of contaminated drinking water. District RRT visited the area and investigated the outbreak. Medical camp organized, chlorination of water supplies carried out. Medical camp organized and all cases treated symptomatically. Garbage removal done from near water sources. Health education was given regarding proper purification of drinking water, hand washing, and food hygiene. 1 stool sample collected and sent to KAPV Medical College, Trichy. The sample tested positive for V.cholerae by culture. Chlorination in all the drinking water sources done. |
| 19 | 2012 | West Bengal | Bankura | Cholera | 56 | 0 | 5/9/2012 | 5/10/2012 | Under surveillance | Cases of diarrhea and vomitting reported from Village Bhatpukur, SC-Ramsagar, GP Ramsagar, Block Onda, District Bankura. District RRT visited the area and investigated the outbreak. It was found that the sanitation level in the area was poor and villagers were using the same water source for drinking as well as washing. 5 stool samples collected and sent to NICED, Kolkata for further investigation. All samples tested positive for V.cholerae (Ogawa strain) by culture. Water samples collected from 1 tubewell and 1 pond and send to PHE (Public Health Engineering) Lab for MPN count. Both results are awaited. Anti- diarrheal medicine distributed in the area. Disinfection of water sources done. IEC done and health education imparted. |
| 21 | 2012 | Punjab | SAS Nagar | Cholera | 15 | 0 | 5/22/2012 | 5/24/2012 | Under control | Cases reported from Village Dappar, PHC Derabassi, District SAS Nagar possibly due to contamination of drinking water. District RRT investigated the outbreak. 3 stool samples were positive for Cholera culture. IEC activities done. |
| 22 | 2012 | Karnataka | Tumkur | Cholera | 59 | 0 | 5/25/2012 | 5/25/2012 | Under Control | Outbreak reported from Villages Gummanahalli, Gollarahatti, PHC Kallambella, Block Sira, District Tumkur. District RRT investigated the outbreak. Out of 5 stool samples collected 1 sample was positive for cholera culture. All cases treated. Chlorination of water sources done. Health education given. |
| 22 | 2012 | Assam | Darrang | Cholera | 38 | 1 | 5/16/2012 |  | Under Control | Outbreak reported from Village Athaiabari, SC Namkhola, Block Patharighat, District Darang. District RRT investigated the outbreak. Out of 2 stool sample collected 1 was positive of cholera culture. House to house survey done for finding new cases. All cases treated locally. Health education given. |
| 22 | 2012 | Karnataka | Mandya | Cholera | 25 | 0 | 5/19/2012 |  | Under Control | Outbreak reported from Village Dodderi Ganappanahalli, District Mandya. District RRT investigated the outbreak. 2 Stool samples collected, were positive for cholera culture. House to house survey done. All cases treated. Alternate drinking water arranged. Temporary clinics organized. Health Education given. IEC given at Grampanchayath level. |
| 22 | 2012 | Maharashtra | Dhule | Cholera | 26 | 0 | 5/15/2012 |  | Under Control | Outbreak reported from Village Khambale, PHC Rohini, Taluka Shirpur, District Dhule. District RRT investigated the outbreak. It was found that due to natural calamity electricity supply of village was cut off for few days so people didn’t had access to safe drinking water. 2 stool samples taken, out of which 1 tested positive for *V.cholerae* by culture. Electricity supply was restored and pipeline leakages corrected. Mass survey conducted and health education given. |
| 23 | 2012 | Assam | Dima Hasao | Cholera | 322 | 6 | 6/5/2012 | 6/10/2012 | Under surveillance | Outbreak reported from Villages Pedik, Daobilidisa, Khepre, Langbrobra, Thonggibra, Diyangbra, Langbrobru and Wajao of Sub-Centre’s Pedik, Bankai, Khepre and Wajao under CHC Maibong & BPHC Langting in District Dima Hasao. The villages are in hilly terrain area and bordering the state of Nagaland. People in these villages use untreated stream water which is likely to be contaminated upstream. All age group are equally affected. Medical team deployed from Maibong CHC. Water sample collected and tested by H2S test and MPN test showed fecal contamination. 2 out of 4 stool samples collected and sent to Microbiology Deptt. GMCH tested positive for Cholera. 108 emergency services are engaged for transportation of patients. IEC activities done. Active search is being done by paramedical staff for new cases. |
| 23 | 2012 | Tamil Nadu | Virudhunagar | Cholera | 13 | 1 | 6/9/2012 | 6/10/2012 | Under control | Cases reported from Village Thiruvirunthalpuram, HSC Amanakkunatham, PHC Malaipatti, Block Aruppukottai, District Virudhunager possibly due to consumption of contaminated water. District RRT visited the area and investigated outbreak. One patient had reportedly become critical and lost consciousness. The patient expired as she was being shifted to a Private Hospital in Aruppukottai. House to house surveillance conducted along with distribution of ORS packets. 1 stool sample collected and sent to Microbiology Department, Madurai Medical College. The same tested positive for *V.cholerae* by culture. 4 water samples collected and sent to Chief Water Analyst, King Institute, Guindy. Results awaited. Chlorination of drinking water being done. Pipelines repaired. Health Education given to the community regarding drinking of boiled and chlorinated water. |
| 24 | 2012 | Karnataka | Mandya | Cholera | 41 | 0 | 6/10/2012 | 6/14/2012 | Under control | Outbreak reported from Village Lakshmi Sagara, PHC/CHC Kerethonur, Block Pandavapura, District Mandya. District RRT investigated the outbreak. 2 samples collected were positive for Cholera culture. House to house survey done. Temporary clinics established for treating cases. Alternate source of drinking water supplied. Health Education given. I E C done at village level. |
| 25 | 2012 | Assam | Kamrup ( Metro) | Cholera | 34 | 0 | 6/18/2012 | 6/22/2012 | Under control | Outbreak reported from Village Gumaria, SC Gumaria, PHC Hahara, Block Sonapur, District Kamrup (Metro). Medical team investigated the outbreak. House to house survey done by paramedical staff. 1 Stool sample collected was positive for Cholera culture. Temporary medical camps organized. All cases treated. Halogen tablet, ORS and anti-diarrhoeal medicines were distributed. Water sources disinfected by PHE dept. |
| 25 | 2012 | Haryana | Panchkula | Cholera | 59 | 0 | 6/13/2012 | 6/23/2012 | Under control | Outbreak reported from Village Budanpur, SC Abeypur, PHC Old Panchkula, CHC Kalka, District Panchkula possibly due to consumption of contaminated drinking water. District RRT investigated the outbreak. 4 water samples collected; all of which were found fit for drinking. 20 stool samples taken and sent to General Hospital, Panchkula. Out of these, 7 samples tested positive for V.cholerae by culture. Alternative water supply arranged. Medical posts established in the area. Health education given. |
| 25 | 2012 | Karnataka | Belgaum | Cholera | 141 | 0 | 6/22/2012 | 6/23/2012 | Under control | Outbreak reported from Village Shukrawarpete area (Sambra), PHC/CHC Mutaga, Block and District Belgaum. District RRT team investigated the outbreak. 6 stools samples collected, 5 were positive for cholera culture. Mobile clinics were established. All cases treated locally. Alternate safe drinking water source provided. Halogen tablets and ORS distributed in the community. Chlorination of drinking water supply done. IEC done. |
| 25 | 2012 | Chandigarh | Chandigarh | Cholera | 5 | 0 | 6/4/2012 |  | Under control | Cases reported from area Manimajra, Chandigarh. District RRT visited the area and investigated the outbreak. Cases tested positive by Hanging Drop and were confirmed by culture. Instructions sent to public health laboratories for water testing and to report chlorine content and coliform count. Health education and IEC done. |
| 25 | 2012 | Puducherry | Puducherry | Cholera | 5 | 0 | 6/11/2012 |  | Under control | Cases reported from1 Thavalakuppam PHC coverage area of Puducherry. RRT investigated the outbreak. Samples tested positive for *V.cholerae* in Arupadaiveedu Medical College, Mahatma Gandhi Medical College and JIPMER. RRT talking all necessary steps for diagnosis of any fresh cases and also taking all corrective measures including chlorination of OHT. Department of Health and IDSP are also taking all steps. IEC conducted. |
| 26 | 2012 | Chandigarh | Chandigarh | Cholera | 6 | 0 | 6/20/2012 | 6/20/2012 | Under control | Cases reported from urban area Vikas Nagar, Maulijagran, Chandigarh. District RRT visited the area and investigated the outbreak. Cases tested positive by Hanging Drop and were confirmed by culture. Health education and IEC done. |
| 26 | 2012 | Karnataka | Chitradurga | Cholera | 18 | 0 | 6/21/2012 | 6/22/2012 | Under control | Outbreak reported from Village Gollanakatte, SC J.N.Kote, District Chitradurga. District RRT investigated the outbreak. 1 stool sample collected and tested positive for Cholera culture. House to house survey done. All cases treated. Health education given. |
| 26 | 2012 | Karnataka | Bangalore Urban | Cholera | 9 | 0 | 6/23/2012 | 6/25/2012 | Under control | Cases reported from Village and PHC/CHC Gopalapura, Block Bangalore North, District Bangalore Urban. District RRT investigated the outbreak. 1 Stool sample collected and was tested positive for Cholera culture. House to House active search for cases done. Disinfection of the surrounding done. Chlorination of drinking water sources done. Halogen tablets and ORS distributed in the community. Awareness of drinking boiled water done at household level. Health Education given. |
| 26 | 2012 | Karnataka | Bidar | Cholera | 23 | 0 | 6/27/2012 | 7/1/2012 | Under control | Outbreak reported from Village Gadi Raipalli(Kadepur), PHC Muchlam, Block Basawakalyan, District Bidar. District RRT investigated the outbreak. Out of 7 stool samples collected, 3 samples were positive for Cholera culture. Pipelines were repaired, OHT cleaned and disinfected. Halogen tablets distributed. Disinfection of houses done & IEC regarding personal hygiene imparted. |
| 26 | 2012 | Kerala | Wayanad | Cholera | 7 | 1 | 6/23/2012 | 6/30/2012 | Under control | Cases reported from Village Noolpuzha, SC Kallor, PHC Noolpuzha, Block Pulpally, District Wayanad. District RRT investigated the outbreak. House to house survey done. Out of 2 stool samples collected 1 was positive for Cholera culture. All the cases were in the age group above 30yrs. Medical camps organized in the affected area. Chlorination of water sources done. ORS packets distributed. Health education done. |
| 26 | 2012 | Haryana | Yamuna Nagar | Cholera | 27 | 0 | 5/29/2012 |  | Under surveillance | Cases of diarrhea and vomiting reported from Village Dhalor, SC Chaharwala, PHC Haibatpur, CHC Bilaspur, District Yamuna Nagar. District RRT visited the area and investigated the outbreak. 27 samples collected, out of which 2 tested positive for Cholera by culture. Chlorination of water sources done. Health education given. |
| 26 | 2012 | Rajasthan | Tonk | Cholera | 35 | 0 | 6/6/2012 |  | Under control | Outbreak reported from Village Kasir, PHC Rajmahal, Block Deoli, District Tonk. District RRT investigated the outbreak. Water samples and 3 stool samples taken for testing. All water samples were found to be potable. Out of 3 stool samples, 1 tested positive for cholera by culture. Health education given to patients regarding use of boiled and chlorinated water. |
| 27 | 2012 | Gujarat | Banaskantha | Cholera | 468 | 5 | 7/2/2012 | 7/3/2012 | Under surveillance | Outbreak reported from Village Dhanera, SC Dhanera PHC Jadiya, Block Dhanera, District Banaskantha. District RRT investigated the outbreak. Out of 9 stool samples collected, 2 were positive for Cholera culture. All age group were affected. Chlorination of OHT done in the community. Pipelines searched for any disruption or leakage. Chlorine tablets distributed in the community. |
| 27 | 2012 | Gujarat | Navsari | Cholera | 87 | 0 | 7/8/2012 | 7/8/2012 | Under surveillance | Outbreak reported from Village Mithilanagri, PHC Chovisi, Block Navsari, District Navsari. Medical team investigated the outbreak. Out of 29 stool samples collected, 3 were positive for Cholera culture. House to house survey done. All cases treated locally. Chlorination of water sources done. ORS packets distributed in the community. IEC activities regarding safe drinking water and healthy sanitation practices done. |
| 27 | 2012 | Haryana | Kaithal | Cholera | 76 | 0 | 6/30/2012 | 7/6/2012 | Under control | Outbreak reported from Urban Area Shakti Nagar, Kaithal city, District Kaithal. District RRT visited the affected area. On investigation it was found that there was mixing of drinking water with sewer water due to leakages in the water supply pipe line. 30 water samples collected for O.T. testing out of which 3 samples were found negative for residual chlorine. 9 stool samples collected and sent to Public Health Lab, Karnal out of which 5 samples tested positive for *V.cholerae* by culture. House to house survey done. Alternate drinking water supply provided. Health camp organized in the affected area and treatment of all patients done. Chlorine tablets and ORS packets distributed. Education about healthy eating habits done. |
| 27 | 2012 | Punjab | SAS Nagar | Cholera | 7 | 0 | 7/4/2012 | 7/6/2012 | Under surveillance | Cases reported from Village Balongi, PHC Gharuan, District SAS Nagar due to consumption of contaminated water (pipeline leakage). District RRT investigated the outbreak. Treatment of patients done at GMCH Mohali. 7 stool samples collected which tested positive for *V*.*cholera* by culture at District Priority Lab (IDSP), Mohali. Chlorine tablets distributed in the locality. IEC activities done regarding use of boiled drinking water. |
| 27 | 2012 | Punjab | Gurdaspur | Cholera | 50 | 0 | 7/1/2012 | 7/5/2012 | Under control | Outbreak reported from Villages Gunopur & Saidowal, CHC Kahnuwan, District Gurdaspur. Block health Team visited the affected villages. Treatment of patients done locally. 9 stool samples collected out of which 4 samples tested positive for *V.cholerae* by culture. Chlorine tablets and ORS packets distributed in the affected villages. IEC activities done regarding safe drinking water and hygiene maintenance. |
| 27 | 2012 | Tamil Nadu | Tirunelveli | Cholera | 27 | 0 | 7/3/2012 | 7/3/2012 | Under control | Outbreak reported from Kadayanallur Municipality, Ward No: 2,4,6,11,31,32,33, District Tirunelvelii, due to contamination of water supply with sewage. Medical team investigated the outbreak. 3 Stool culture collected and sent to TVMCH which tested positive for cholera by culture. Chlorination of OHT done. Surrounding of the houses was disinfected. Health education given. |
| 28 | 2012 | Assam | Cachar | Cholera | 10 | 1 | 7/8/2012 | 7/13/2012 | Under control | Cases reported from Village and SC Bahadurpur, PHC Lakhipur, District Cachar. The district was affected with floods. District RRT investigated the outbreak. Out of 2 stool samples collected, 1 stool sample was positive for cholera culture. ORS and halogen tablets distributed in the community. IEC activities done. |
| 28 | 2012 | Karnataka | Bijapur | Cholera | 32 | 0 | 7/8/2012 | 7/12/2012 | Under control | Outbreak reported from Village/PHC/CHC Talikoti, Block Muddebihal, District Bijapur. District RRT investigate the outbreak. 6 water samples and 2 stools samples collected and sent to District Laboratory. 2 stool samples were positive for cholera culture. Safe drinking water supplied through tankers. Houses to house survey done. Halogen tablets & ORS packets were distributed. Health education regarding personal hygiene imparted. |
| 28 | 2012 | West Bengal | Hoogly | Cholera | 37 | 0 | 7/10/2012 | 7/12/2012 | Under control | Outbreak reported from Village Pandua, Block Golagori, District Hoogly. Block RRT team visited and investigated the outbreak. 4 rectal swab samples collected out of which 1 sample tested positive for cholera by culture at NICED Kolkata. Ten ponds and seven tubewell disinfected. All cases treated. ORS packets and chlorine tablet distributed in the locality. IEC activities undertaken regarding safe food and drinking water, hygienic practices and sanitation. |
| 28 | 2012 | West Bengal | Birbhum | Cholera | 53 | 0 | 7/6/2012 | 7/10/2012 | Under control | Outbreak reported from Village Nowadanga, GP Albandha, Block Bolpur, District Birbhum due to consumption of pond water. District & Block health team investigated the outbreak. 4 rectal swab samples collected and sent to NICED, Kolkatta out of which 1 sample tested positive for cholera by culture. Health education given. |
| 28 | 2012 | West Bengal | Birbhum | Cholera | 21 | 0 | 7/6/2012 | 7/12/2012 | Under control | Outbreak reported from Village & SC Purandarpur, Block Suri-II, District Birbhum due to consumption of contaminated water by pipeline leakage. District & Block health team investigated the outbreak. 2 rectal swab samples collected and sent to NICED, Kolkata out of which 1 sample tested positive for cholera by culture. House to house survey done. Treatment provided to all cases. Disinfection of ponds and ghat done. Health education given regarding use of safe drinking water. |
| 29 | 2012 | Assam | Dibrugarh | Cholera | 15 | 0 | 7/16/2012 | 7/21/2012 | Under control | Outbreak reported from Village Kajonibari, SC Greki Nepali, PHC Panitola, District Dibrugarh. District RRT investigated the outbreak. 4 stool samples collected and sent to Microbiology Dept. AMCH, Dibrugarh, result showed growth of *Virbrio cholerae* (O1 Ogwa). 2 water samples collected from drinking water sources for MPN test and sent to Microbiology Deptt. AMCH, Dibrugarh, result found unsatisfactory. Active search done for finding new cases. PHE Deptt intimated to provide safe drinking water in the affected area. IEC done. |
| 29 | 2012 | Gujarat | Anand | Cholera | 52 | 0 | 7/12/2012 | 7/16/2012 | Under control | Outbreak reported from Village Panchavati, PHC Karamshad, Block Anand, District Anand. Dsitrict RRT investigated the outbreak. Three pipeline leakages were found in affected area. Active surveillance done by PHC staff. District and Block RRT investigated the outbreak. Two stool samples collected and sent to Karamsad Medical College (Anand), tested positive for Cholera. Chlorine tablets and ORS packets distributed. All pipeline leakages repaired and all preventive measures including health education done. |
| 29 | 2012 | Karnataka | Chikmagalur | Cholera | 19 | 0 | 7/21/2012 | 7/21/2012 | Under control | Outbreak reported from Village SG Koppalu, PHC/CHC Jignehalli, Block Kadur, District Chikmagalur. District RRT investigated the outbreak. Active search of new cases done. 2 stool samples collected and sent to Shimoga Medical College; result showed positive for Cholera culture. Safe drinking water was provided in the affected area. Temporary clinic organized in the village. Patients were treated at Kadur General Hospital. |
| 29 | 2012 | Karnataka | Kodagu | Cholera | 12 | 0 | 7/16/2012 | 7/18/2012 | Under control | Cases reported from Village Kurchi Beruga, PHC/ CHC Srimangala, Block Virajpet, Distrcit Kadagu. District RRT investigated the outbreak. Out of 3 stool samples collected, 2 were positive for Cholera culture. |
| 29 | 2012 | Maharashtra | Akola | Cholera | 47 | 0 | 7/18/2012 | 7/19/2012 | Under control | Outbreak reported from Villages Barshitakli, Redwa SC Kajleshwar PHC Kanheri, District Akola. District RRT investigated the outbreak. 7 stool samples were collected of which 2 tested positive for *Vibrio cholerae* at District Public Health Lab Akola. House to house survey done. Chlorination of water sources done. IEC activities done in the affected area. |
| 29 | 2012 | Odisha | Berhampur | Cholera | 23 | 1 | 7/15/2012 | 7/22/2012 | Under control | Outbreak reported from Village Bomokei, GP Bomokei, Block Digapahandi, District Berhampur. Block RRT team investigated the outbreak. Patients were admitted in CHC, Bomokei for treatment. House to house survey done by health staff. Out of 2 stool samples collected, 1 sample was positive for cholera culture. ORS and halogen tablets distributed in the community. Health education given to all affected household regarding personal hygiene such as hand wash, disposal of excreta and safe food handling. |
| 30 | 2012 | Assam | Tinsukia | Cholera | 15 | 1 | 7/18/2012 | 7/25/2012 | Under control | Cases reported from Village Bisakopee TE Line No. 2,3, CHC Domdooma, BPHC Kakopathar, District Tinsukia, due to consumption of contaminated drinking water (Tubewell). District RRT investigated the outbreak. All age group were affected. One stool sample collected and sent to Microbiology Deptt. AMCH, Dibrugarh for culture sensitivity. Result showed growth of Virbro Cholera O1 ogawa positive. Halogen tablets distributed in the community. IEC done. |
| 30 | 2012 | Punjab | Patiala | Cholera | 35 | 0 | 7/23/2012 | 7/29/2012 | Under control | Outbreak reported from Village Bhootgarh, PHC Shutrana, District Patiala due to mixing of drainage water with house hold water supply. District RRT investigated the outbreak. Medical camp organized and medicine distributed in the affected area. 4 Stool samples collected and sent to GMC, Patiala out of which 1 sample tested positive for *Vibrio cholerae* by culture. ORS packets and chlorine tablets distributed. Alternate source of drinking water provided through tankers. IEC activities done. |
| 30 | 2012 | Punjab | Hoshiarpur | Cholera | 45 | 0 | 7/24/2012 | 7/25/2012 | Under control | Outbreak reported from Village Dasua Ward no. 11,12,13, PHC & Block Dasua, District Hoshiarpur due to consumption of contaminated water (pipeline leakage). District RRT investigated the outbreak. 10 Stool samples collected and sent to CMC, Ludhiana out of which 8 samples tested positive for *Vibrio cholerae* by culture. Chlorine tablets distributed. Awareness about general cleanliness, drinking water after boiling was imparted in the community. |
| 30 | 2012 | West Bengal | Hooghly | Cholera | 24 | 0 | 7/26/2012 | 7/27/2012 | Under control | Outbreak of loose motion reported from Municipal Areas Inchhura bazar, Bhaklipara, Gangadharpur and Bakulia, GP Dhabapara, Block Balagarh, District Hooghly. Block health team investigated the outbreak. 3 rectal swab samples collected and sent to NICED, Kolkata out of which 2 samples tested positive for *Vibrio cholerae* by culture. ORS packets and chlorine tablets distributed. Disinfection of ponds and tubewell done in the affected area. IEC activities undertaken regarding safe food and drinking water, hygienic maintenance and sanitation. |
| 30 | 2012 | West Bengal | Bankura | Cholera | 73 | 0 | 7/22/2012 | 7/23/2012 | Under control | Outbreak of loose motion reported from Village Barmagura (Dompara), SC Ramsagar, Block Onda, District Bankura possibly due to consumption of contaminated pond water. District RRT & Block health team investigated the outbreak. Medical treatment provided to all cases. 3 stool samples (rectal swab) collected and sent to BSMCH, Bankura out of which 1 sample tested positive for *Vibrio cholerae* by culture and 2 samples tested positive for *E.coli*. Disinfection of ponds done. Health education given regarding use of safe drinking water. |
| 30 | 2012 | West Bengal | Bankura | Cholera | 54 | 1 | 7/26/2012 | 7/27/2012 | Under control | Outbreak of loose motion reported from Village & SC Kumidya, Block Bankura-I, District Bankura due to consumption of contaminated pond water. District & Block health team visited and investigated the outbreak. Treatment of patients done at BSMCH, Bankura. 4 rectal swab samples collected and sent to BSMCH, Bankura out of which 3 samples tested positive for *Vibrio cholerae* by culture and 1 sample tested positive for *E.coli*. Medicines provided to all cases. Disinfection of ponds done. IEC done. |
| 30 | 2012 | Maharashtra | Parbhani | Cholera | 174 | 1 | 7/17/2012 |  | Under control | Outbreak reported from Village Ratnapur PHC Rampuri Block Manwat due to consumption of contaminated water. District RRT investigated the outbreak. House to house survey done. Out of 57 water samples collected and tested, 23 samples were not potable. Out of 23 stool samples collected at Rural Hospital Manwat and sent to Ratnapur, District Public Health Laboratory (DPHL), 6 samples were tested positive for Vibrio Cholera. ORS distribution and contact treatment given. Health education given. |
| 31 | 2012 | Karnataka | Belgaum | Cholera | 17 | 0 | 8/3/2012 | 8/4/2012 | Under control | Cases reported from Village Thirtkunde, PHC Kanakumbhi, Block Khanapur, District Belgaum. Distrcit RRT investigated the outbreak. Out of 3 stool sample collected, 1sample was positive for Cholera culture. Alternative safe drinking provided in the affected village. Halogen tablets and ORS packets distributed in the community. IEC done. |
| 32 | 2012 | Karnataka | Uttar Kannada | Cholera | 20 | 0 | 8/7/2012 | 8/9/2012 | Under control | Outbreak reported from Villages Yadoga & Ramapur, PHC/CHC Yadoga, Block Haliyal due to contaminated water. All 4 stool samples collected were positive for Cholera culture. Halogen tablets distributed in the community. . |
| 32 | 2012 | Odisha | Nuapada | Cholera | 17 | 1 | 8/10/2012 | 8/10/2012 | Under control | Cases reported from Village Jambahali, CHC Boden, Distrcit Nuapada. Block health team investigated the outbreak. All cases treated locally. 3 stool samples collected and sent to State Lab out of which 1 sample tested positive for cholera. Chlorine tablets distributed in the community. IEC activities done. |
| 32 | 2012 | Punjab | Hoshiarpur | Cholera | 80 | 0 | 8/5/2012 | 8/6/2012 | Under control | Outbreak reported from Village Chak Mehra, PHC Mand Mander, District Hoshiarpur due to consumption of contaminated water. District RRT investigated the outbreak. 51 cases treated at medical camp organized in the locality. 29 patients treated at Civil Hospital, Dasuya. 5 stool samples collected and sent to CMCH Ludhiana out of which 3 samples tested positive for cholera by culture. 8 water samples collected and sent to SPHL Chandigarh out of which 5 samples were non potable. Chlorine tablets distributed. Awareness given to local people about cleanliness and use of boiled water for drinking purpose. |
| 33 | 2012 | West Bengal | Jalpaiguri | Cholera | 19 | 0 | 8/17/2012 | 8/18/2012 | Under control | Cases reported from Village Chattandoo, Block Nagrakata, District Jalpaiguri due to consumption of contaminated water. District RRT & Block health team investigated the outbreak. Active search for cases done. People practice open defecation. Well is the main source of drinking water in the village. Symptomatic treatment provided and ORS packets given to cases. 12 patients treated at local health centre. 3 stool samples collected and sent to NBMCH Darjeeling out of which 2 samples tested positive for *Vibrio cholerae*. Chlorination of water sources done. IEC done. |
| 33 | 2012 | West Bengal | Birbhum | Cholera | 19 | 0 | 8/6/2012 |  | Under control | Cases reported from Village Domonpur, GP Dharampur, SC Gopinathpur, PHC & Block Illambazar, District Birbhum due to consumption of contaminated water (pipeline leakage). District RRT and Block health team investigated the outbreak. House to house survey done by health workers. All cases treated symptomatically. 1 rectal swab sample collected and sent to NICED, Kolkata which tested positive for *Vibrio Cholerae* (O1 Ogawa). Halogen tablets and ORS packets distributed. Disinfection of ponds, ghat and well done. IEC done. |
| 34 | 2012 | Haryana | Jind | Cholera | 119 | 0 | 8/23/2012 | 8/26/2012 | Under control | Outbreak reported from Village & SC Bharmanwas, CHC Julana, District Jind. District RRT investigated the outbreak. On investigation it was found that there was mixing of sewer water with drinking water supply. Open drainage system was present in the village. House to house survey done. Health camp established in the locality. 3 stool samples collected and sent to PHL Karnal out of which 1 sample tested positive for *Vibrio cholerae* and *Klebsiella pneumoniae* and 2 samples were positive for *E. coli* by culture. Halogen tablets and ORS packets distributed. Chlorination of wells done. Alternate supply of drinking water provided. Health education given to the local people regarding use of safe water for drinking purpose. |
| 34 | 2012 | Karnataka | Bijapur | Cholera | 85 | 0 | 8/20/2012 | 8/20/2012 | Under control | Outbreak reported from Village/PHC Harnal, Block Muddeebihal, District Bijapur due to consumption of contaminated well water. District RRT investigated the outbreak. Out of 5 stool samples collected, 3 samples were positive for cholera. Chlorination of water sources done. Halogen tablet distributed in the community. IEC done. |
| 34 | 2012 | Maharashtra | Nandurbar | Cholera | 18 | 0 | 8/19/2012 | 8/22/2012 | Under control | Cases reported from Village/SC Dhudhkheda, PHC Shahana, Block Shahada, District Nandurbar, due to consumption of contaminated water. District RRT investigated the outbreak. House to house survey done. 4 stool samples collected which tested positive for cholera culture. Chlorination of water sources done. Health education given regarding safe drinking water and sanitation. |
| 35 | 2012 | Haryana | Karnal | Cholera | 26 | 0 | 9/1/2012 | 9/2/2012 | Under control | Outbreak reported from Village Bal Ragran, PHC Gagsina, CHC Ghraunda, District Karnal due to consumption of contaminated water (pipeline leakage). District RRT investigated the outbreak. House to house survey done. Halogen tablets distributed by health workers. ORS packets given to cases. 8 stool samples collected and sent to State Public Health lab Karnal out of which 5 samples tested positive for *Vibrio cholerae*. Repair of pipeline leakages done by village health & sanitation committee. Alternate source of drinking water provided in the community. People were advised to use boiled water for drinking purpose. |
| 36 | 2012 | Punjab | Patiala | Cholera | 17 | 1 | 9/4/2012 | 9/8/2012 | Under control | Cases reported from Village Rampura Patran (Valmiki Basti), PHC Shutrana, District Patiala due to consumption of contaminated water (pipeline leakage). District RRT investigated the outbreak. Medical camp organized in the affected area. Chlorine tablet and ORS packets distributed in the locality. 5 stool samples collected and sent to GMC Patiala out of which 3 samples tested positive for Vibrio cholera (O1 Ogawa). Alternate supply of drinking water provided in the community. IEC done regarding safe drinking water, hygiene and sanitation. |
| 36 | 2012 | West Bengal | Bankura | Cholera | 90 | 0 | 9/8/2012 | 9/9/2012 | Under control | Outbreak of loose motion reported from Village Fulberia, SC Nabajibanpur, Block Onda probably due to consumption of contaminated pond water. District RRT along with Block health team visited the affected area. People practice open defecation and use pond water for daily activities. All cases treated locally. 3 rectal swab samples collected and sent to BSMCH Bankura which tested positive for Vibrio cholera (O1 Ogawa). Disinfection of water sources done. Health education imparted to the people regarding use of safe water for drinking purpose. |
| 36 | 2012 | Maharashtra | Nagpur | Cholera | 161 | 0 | 8/27/2012 |  | Under control | Outbreak reported from Village Nilaj, PHC Kanhan, Block Parshioni, District Nagpur. Medical team investigated the outbreak. All cases treated. House to house survey done. Out of 4 stool samples collected 1 sample was positive for cholera culture. Disinfection of surrounding done. Chlorination of water sources done Pipelines was repaired. |
| 36 | 2012 | West Bengal | Jalpaiguri | Cholera | 23 | 0 | 8/25/2012 |  | Under control | Outbreak reported from SC’s Sulkapara & Khairbari, Block Nagrakata, District Jalpaiguri. District RRT and Block health team investigated the outbreak. Active search for cases done. People practice open defecation. All cases treated locally. ORS packets given to cases. 5 stool samples collected and sent to NBMCH Darjeeling which tested positive for *Vibrio cholerae*. Chlorination of water sources done. IEC activities done. |
| 37 | 2012 | Odisha | Rayagada | Cholera | 37 | 3 | 9/11/2012 | 9/16/2012 | Under control | Outbreak reported from Villages Katiguda & Purtiguda, SC Dhepaguda & Madhuban, Block Gudari, Distrcit Rayagada. Medical team investigated the outbreak. All cases treated. Control room established at Dhepaguda. Out of 19 rectal swabs collected, 18 were positive for Cholera culture. Chlorination of water sources done. Chlorine tablets distributed. Health education given. |
| 38 | 2012 | Jammu & Kashmir | Baramulla | Cholera | 238 | 0 | 9/19/2012 | 9/23/2012 | Under control | Outbreak of loose motion and vomiting reported from Villages Ferozpora, Drung, Mahin, Killwara, Check, Hariwatnu and Qazipora. Block Tangmarg, District Baramulla. State RRT visited the affected area. On investigation it was observed that all the affected villages had a common source of water supply from a reservoir in Village Drung which was faecally contaminated. Health camp established in the community. 238 cases were given symptomatic treatment. 5 rectal swab samples collected and sent to PHL Srinagar out of which 1 sample tested positive for *Vibrio cholerae* (O1 Ogawa). 21 patients treated at SKIMS Srinagar. Out of 21 stool samples tested at SKIMS Srinagar, 6 samples were positive for *Vibrio cholerae* (O1 Ogawa). 6 water samples collected and tested for MPN count were found contaminated and unsatisfactory for drinking purpose. Alternate supply of drinking water provided by PHED. Chlorine tablets distributed to all households. Disinfection of surrounding done. Health education given regarding use of boiled water for drinking purpose and washing of hands after using toilet and before eating food. |
| 38 | 2012 | Karnataka | Uttara Kannada | Cholera | 66 | 0 | 9/12/2012 | 9/13/2012 | Under control | Outbreak reported from Village Gunjavathi & Manalli, PHC/CHC Arshangeri, Block Mundgod, District Uttara Kannada. Block Medical team investigated the outbreak. Out of 10 stool samples collected 1 sample was positive for cholera culture. Control measures undertaken. |
| 38 | 2012 | Karnataka | Koppal | Cholera | 16 | 0 | 9/19/2012 | 9/23/2012 | Under control | Cases reported from Village Handral, PHC/CHC Alwandi, Block Koppal due to consumption of contaminated water. District RRT investigated the outbreak. All cases treated locally. Out of 4 stool samples collected 1 sample was positive for cholera culture. Chlorination of water sources done. Health education given. |
| 38 | 2012 | West Bengal | Malda | Cholera | 87 | 0 | 9/19/2012 | 9/23/2012 | Under control | Outbreak of loose motion and vomiting reported from Village Naldubi, Block Old Malda, District Malda. District RRT & Block health team investigated the outbreak. The sanitary condition was very poor in the affected village. People consumed water from tube well and pond. Medicine and ORS packets given to cases. 7 rectal swab samples collected and sent to STM Kolkata out of which 2 samples tested positive for *Vibrio cholerae* by culture. Chlorine tablets distributed in the locality. Disinfection of tube wells done by PHED. People advised to stop consumption of pond water. IEC activities done. |
| 38 | 2012 | West Bengal | Hooghly | Cholera | 65 | 2 | 9/10/2012 |  | Under control | Outbreak of loose motion reported from Chapdani Municipality (Ward No 3, 4, 5 & 19), District Hooghly. District RRT investigated the outbreak. House to house survey done. Cases treated at local health centre. 3 rectal swab samples collected and sent to NICED Kolkata out of which 1 sample tested positive for *Vibrio cholerae* (O1 Ogawa). ORS packets and Chlorine tablets distributed in the community. IEC activities undertaken regarding safe food and drinking water, hygiene and sanitation. |
| 40 | 2012 | Assam | Barpeta | Cholera | 13 | 0 | 9/30/2012 | 10/5/2012 | Under Control | Cases reported from Village & SC Fulora and Village Chapari Pathar, SC Mawamari, PHC Mandia, District Barpeta. District RRT investigated the outbreak. House to house survey done. Symptomatic treatment provided to cases. 3 stool sample collected and send to Guwahati Medical College & Hospital, out of which 1 sample tested positive for *Vibrio Cholerae.* ORS packets and Halogen tablets distributed in the locality. IEC done regarding safe drinking water and personal hygiene. |
| 40 | 2012 | Assam | Jorhat | Cholera | 84 | 0 | 9/28/2012 | 10/3/2012 | Under Control | Outbreak reported from Villages Gojpur and Barhoi Bari, PHC & Block Baghchang, District Jorhat. District RRT investigated the outbreak. House to house survey done. Medical camp organized in the affected area. 3 stool samples collected and sent to Jorhat Medical College & Hospital, out of which 1 sample tested positive for *Vibrio Cholerae*. Further 2 stool samples collected; result awaited. 6 water samples collected out of which 3 samples were faecally contaminated. Medicine and ORS packets given to cases. Halogen tablets distributed in the community by paramedical staff. Public Health Engineering Department informed to provide safe drinking water. Health education given. |
| 40 | 2012 | Punjab | Gurdaspur | Cholera | 383 | 18 | 10/5/2012 | 10/6/2012 | Under surveillance | Outbreak of gastroenteritis reported from areas of Gandhi Nagar camp, Murgimohalla, Longowal and Gurunanak Nagar in Batala Industrial Town, District Gurdaspur. State/District RRT investigated the outbreak. House to house survey done. Water pipeline leakages along with sewage bocks at multiple points were observed in the affected area. Temporary Medical camp established in the locality. Cases treated locally and at Civil Hospital Batala. A Central team from NCDC Delhi visited the affected area. Five out of seven stool samples that were processed in Civil Hospital, Batala, yielded *Vibrio cholerae* O1 Ogawa. Subsequently, state also reported 6 stool samples positive for *Vibrio cholerae* out of 43 samples sent to Govt. Medical College Amritsar. The water samples collected (12 out of 23) from the water pumps in affected areas tested positive for faecal contamination at State Public Health Lab, Chandigarh. ORS packets & Chlorine tablets distributed in the locality. Leakages in water pipeline repaired by Municipal Corporation Balata. Alternate supply of drinking water provided in the community. Chlorination of water sources done. IEC done. |
| 40 | 2012 | Punjab | Moga | Cholera | 109 | 1 | 10/7/2012 | 10/7/2012 | Under surveillance | Cases of loose motion and vomiting reported from urban area New Dana Mandi, Moga city, District Moga due to consumption of contaminated water (pipeline leakage). District RRT investigated the outbreak. House to house survey done. All cases were migratory labourers. Cases treated at Civil Hospital Moga. 5 stool samples collected and sent to Guru Govind Singh Medical College Faridkot out of which 3 samples tested positive for *Vibrio Cholerae*. 3 patients treated at Guru Govind Singh. Medical College Faridkot. 7 water samples tested at State Public Health laboratory Chandigarh were non potable. ORS packets & Chlorine tablets distributed to the local households. Alternate supply of drinking water provided in the community. Health education given regarding hygiene and sanitation. |
| 42 | 2012 | Karnataka | Belgaum | Cholera | 151 | 2 | 10/17/2012 | 10/18/2012 | Under control | Outbreak reported from Village/PHC Sadalaga, Block Chokkodi, District Belgaum. District RRT investigated the outbreak. House to house survey done. Out of 6 stool samples collected 2 samples were tested positive for Cholera culture. All cases treated. Chlorination of water sources done. ORS and Halogen tablets distributed in the community. IEC done. |
| 44 | 2012 | West Bengal | Bankura | Cholera | 61 | 0 | 11/4/2012 | 11/4/2012 | Under control | Outbreak of loose motion reported from Village Chayanpur, SC Gobindadham, Block Amarkanan, District Bankura. District RRT investigated the outbreak. Active search for cases done. People practice open defecation and consume pond water for cleaning of utensils and drinking purpose. Cases treated at local health centre. 4 rectal swab samples collected and sent to BSMCH Bankura out of which 1 sample tested positive for cholera (O1 Ogawa). Chlorination of water sources done. People advised to stop consumption of pond water. |
| 45 | 2012 | Punjab | Amritsar | Cholera | 17 | 0 | 11/7/2012 | 11/7/2012 | Under control | Cases of loose motion reported from Sant Mishra Singh Colony, Amritsar city, District Amritsar. District RRT investigated the outbreak. House to house survey done. Cases treated locally. 6 stool samples collected and sent to Govt. Medical College Amritsar out of which 3 samples were positive for *Vibrio cholerae* by culture. Chlorine tablets and ORS packets distributed in the community. IEC done. |
| 46 | 2012 | Chhattisgarh | Durg | Cholera | 579 | 1 | 10/30/2012 |  | Under control | Outbreak of loose motion and vomiting reported from Urban areas Kandrapara, Saraswati Nagar, Shivpara, Rajeev Nagar, Panchsheel nagar, Chandi Mandir, Bandha Talaab, Beldarpara, Nawapara, Sarathipara and Bajrang Nagar of Durg city, District Durg. District RRT investigated the outbreak. Temporary medical camp organized in the community. Cases treated symptomatically. Chlorine tablets distributed to the local households. ORS packets given to cases. Out of 37 rectal swab `samples collected and sent to Pt. JNM Medical College Raipur, 4 samples were positive for *V. cholerae* (O1 Ogawa) by culture. Control measures undertaken. |
| 51 | 2012 | Andhra Pradesh | Anantapur | Cholera | 23 | 0 | 12/20/2012 | 12/23/2012 | Under control | Outbreak of loose motion reported from Village Linganahalli, PHC D.Hirehal, District Ananthapur due to consumption of contaminated water (pipeline leakage). District RRT investigated the outbreak. Cases treated symptomatically at health camp organized in the locality. Out of 2 stool samples collected and sent to IPM Hyderabad, 1 sample was positive for cholera. Chlorination of water sources done. Pipeline leakage repaired. Alternate supply of drinking water provided in the community. IEC done regarding water borne diseases. |
|  |  |  |  |  |  |  |  |  |  |  |
| Wk No. | Year | State | District | Disease | Cases | Deaths | Date of Outbreak | Date of Reporting | Current Status | Comments |
| 2 | 2013 | West Bengal | Hooghly | Cholera | 69 | 0 | 12/15/2012 |  | Under control | Outbreak of loose motion, vomiting and abdominal pain reported from Village Bhowpur, SC Jamna, Block Pandua, District Hooghly. District RRT and Block health team investigated the outbreak. Cases treated symptomatically. 28 patients treated at local health centre. 8 rectal swab samples collected and sent to NICED Kolkata out of which 3 samples were positive for *Vibrio cholerae* (O1 Ogawa). Chlorine tablets and ORS packets distributed in the community. IEC activities undertaken regarding safe food and drinking water, hygiene and sanitation. |
| 7 | 2013 | West Bengal | Purulia | Cholera | 195 | 2 | 1/13/2013 |  | Under control | Outbreak of gasteroenteritis reported from town area Purulia, Block/District Purulia due to consumption of contaminated water (pipeline leakage). District RRT investigated the outbreak. Population of the affected area was 25000. A total of 195 cases and 2 deaths occurred during 13^th^ to 30^th^ January 2013. House to house survey done. Cases treated at local health centre. 2 stool samples collected which tested positive for *Vibrio cholerae* (O1 Ogawa). Alternate supply of drinking water provided in the community by Purulia Municipality. Pipeline leakages repaired. Chlorination of water sources done. IEC done regarding safe drinking water, hygiene and sanitation through distribution of pamphlets and miking. |
| 10 | 2013 | Karnataka | Raichur | Cholera | 20 | 0 | 3/4/2013 | 3/10/2013 | Under control | Outbreak of loose motion, vomiting and abdominal pain reported from Village Koppar, PHC/CH Koppar, Block Devdurga, District Raichur. Medical team investigated the outbreak. House to house survey done. Out of 4 stool samples collected, 3 samples were positive for Cholera by culture. IEC activities undertaken regarding safe food and drinking water, hygiene and sanitation. |
| 10 | 2013 | Karnataka | Raichur | Cholera | 10 | 0 | 3/4/2013 | 3/10/2013 | Under control | Outbreak reported from Village/PHC/CHC Masarkal, Block Devdurga, District Raichur. House to house survey done. Out of 2 stool samples collected 1 sample was positive for Cholera by culture. IEC done regarding safe drinking water, hygiene and sanitation. |
| 12 | 2013 | Karnataka | Haveri | Cholera | 72 | 1 | 3/18/2013 | 3/19/2013 | Under control | Outbreak reported from Village Belavigi, PHC/CHC Negalur, Block & District Haveri. District RRT investigated the outbreak. House to house survey done. Out of 9 stool samples collected, 6 samples were positive for cholera by culture. ORS packets and chlorine tablets distributed in the community. Chlorination of water sources done. IEC done. |
| 16 | 2013 | Karnataka | Davangere | Cholera | 36 | 0 | 4/19/2013 | 4/21/2013 | Under control | Cases of loose motion reported from Village Kakanuru, PHC Kondadahalli, Block Channagiri, District Davengere. Medical team investigated the outbreak. House to house survey done. Pipeline leakages were observed in the community. Cases treated locally. Out of 6 stool samples collected 5 were positive for cholera by culture. Out of 2 water samples collected, 1 sample was non potable. Chlorination of water sources done. Pipeline leakages repaired. Health education given. |
| 16 | 2013 | Karnataka | Mysore | Cholera | 129 | 0 | 4/13/2013 | 4/18/2013 | Under surveillance | Outbreak of gastroenteritis reported from Village Jakkalli, PHC Hebbalaguppe, Block HD Kote, District Mysore due to consumption of contaminated water (pipeline leakage). District RRT investigated the outbreak. Temporary medical camp established in the locality. Cases treated symptomatically. Out of 15 stool samples collected, 6 samples were positive for *Vibrio cholerae* by culture*.* Alternate supply of drinking water provided in the community. Chlorine tablets and ORS packets distributed. Pipeline leakages repaired. Health education given regarding safe drinking water, hygiene and sanitation. |
| 16 | 2013 | Karnataka | Tumkur | Cholera | 44 | 0 | 4/19/2013 | 4/19/2013 | Under control | Outbreak of loose motion and vomiting reported from Village/ PHC Halkurike, Block Tiptur, District Tumkur. District RRT investigated the outbreak. House to house survey done. Cases treated symptomatically. Out of 3 stool samples collected, 1 sample was positive for *Vibrio cholerae* by culture. Out of 4 water samples collected, 3 samples were non potable. Chlorination of water sources done in the community. Disinfection of the surrounding done. Health education given. |
| 17 | 2013 | Karnataka | Chitradurga | Cholera | 40 | 2 | 4/25/2013 | 4/26/2013 | Under control | Outbreak of loose motion and vomiting reported from Village Talavatti, SC Salabommanahalli, PHC Aimanagala, Block Hiriyur, District Chitaradurga. District RRT investigated the outbreak. House to house survey done. Cases treated. Out of 4 stool samples collected, 2 samples were positive for cholera (O1 ogawa). Chlorination of water sources done by Gram Panchayat. Health education given regarding safe drinking water, hygiene and sanitation. |
| 17 | 2013 | Kerala | Wayanad | Cholera | 18 | 2 | 4/19/2013 | 4/27/2013 | Under surveillance | Outbreak of loose motion reported from Muttil Panchayat (Aduvadi,Thekkumpagy colony & Ambathamvayil tribal colonies), SC Mandad & Kallupady, PHC Vazavata, District Wayanad. Medical team investigated the outbreak. House to house survey done. A total of 18 cases and 2 deaths were reported from 19^th^ to 27^th^ April, 2013. Community consumed stream water and open defecation was rampant. 2 deaths occurred (age 65 yrs and 24 yrs). 1 stool sample collected tested positive for cholera culture. Disinfection of surrounding done. Chlorination of water sources done. ORS packets distributed in the community. Arranged drinking water with help of LSG. Intersectoral coordination meeting organized. IEC done. |
| 18 | 2013 | West Bengal | South 24 Parganas | Cholera | 70 | 0 | 4/30/2013 | 5/5/2013 | Under control | Outbreak of loose motion and vomiting reported from Village Potrakhali, SC Satiakhali, Block Basanti, District Bankura. District RRT investigated the outbreak. House to house survey done. Medical camp organized in the community. Medicine and ORS packets given to cases. Out of 3 stool sample collected 2 samples were positive for cholera culture. IEC done. |
| 19 | 2013 | West Bengal | Purulia | Cholera | 60 | 1 | 5/1/2013 | 5/2/2013 | Under surveillance | Outbreak of loose motion reported from Village Mahultanr, SC Chatuhansa, Block Arsha, District Purulia. District RRT investigated the outbreak. People consumed water from stream and well. People practice open defecation. Cases treated at temporary health camp organized in the community. 1 death occurred in a patient aged 22 years. Out of 5 stool samples collected and sent to BMCH Bankura, 3 samples were positive for *V. cholerae* (O1 ogawa). Disinfection of surrounding done. Chlorination of water sources done. IEC done regarding safe drinking water. |
| 20 | 2013 | Karnataka | Davangere | Cholera | 32 | 0 | 5/14/2013 | 5/14/2013 | Under control | Cases of gastroenteritis reported from Village Hosa Obalpura, SC Bennehalli, Block Harapanahalli, District Davangere. District RRT investigated the outbreak. House to house survey done. Out of 3 stool samples collected, 1 sample was positive for cholera by culture. Out of 4 water samples collected, 3 samples were unfit for drinking purpose. Chlorine tablets were distributed in the community. Chlorination of water sources done. IEC done regarding safe drinking water, hygiene and sanitation. |
| 20 | 2013 | West Bengal | Bankura | Cholera | 70 | 0 | 5/7/2013 |  | Under control | Cases of loose watery stools and vomiting reported from Village Potrakhali, SC Satiakhali, PHC Chunakhali, Block Basanti, District Bankura. District RRT and Block health team investigated the outbreak. House to house survey done. People consumed water from tube well. Symptomatic treatment given to cases. Out of 3 stool samples collected, 1 sample was positive for cholera by culture. Chlorination of water sources done. Health education given regarding safe drinking water. |
| 21 | 2013 | Gujarat | Gandhinagar | Cholera | 87 | 2 | 5/14/2013 |  | Under surveillance | Outbreak of loose motion and vomiting reported from Village Puna Talavadi, PHC/Block Dehgam, District Gandhinagar. District RRT investigated the outbreak. House to house survey done. Pipeline leakages were observed in the community. Cases treated symptomatically. 2 stool samples collected and sent to BJMC Ahmedabad which tested positive for cholera by culture. Chlorine tablets and ORS packets were distributed in the affected area. Pipeline leakages were repaired. Health education given regarding safe drinking water. |
| 21 | 2013 | Maharashtra | Sangli | Cholera | 88 | 0 | 5/5/2013 |  | Under control | Cases of loose motion and vomiting reported from Village Bilashi, SC/PHC Kokrud, Block Shirala, District Sangli. District RRT investigated the outbreak. House to house survey done. Pipeline leakages were observed in the locality. Temporary medical camp organized. Cases treated symptomatically. 2 stool samples collected which tested positive for cholera. Chlorination of water sources done. Pipeline leakages repaired. Health education given regarding use of boiled water for drinking purpose. |
| 22 | 2013 | Goa | North Goa | Cholera | 97 | 1 | 5/30/2013 | 5/31/2013 | Under surveillance | Outbreak of nausea, vomiting and loose motion reported from Aguada Jail, PHC Candolim, District North Goa. District RRT visited the jail premises and investigated the outbreak. Temporary medical camp organized. Medicine given to cases. 35 cases treated at tertiary care hospital. Stool, food and water samples collected; result awaited. Out of 6 stool samples collected, 3 samples tested positive for V. cholerae. Water samples (tap water) collected from Aguada and Mapusa jails were contaminated with coliform organisms. |
| 22 | 2013 | West Bengal | North 24 Parganas | Cholera | 144 | 0 | 5/25/2013 | 5/27/2013 | Under surveillance | Outbreak of abdominal pain, loose motions, vomiting, dehydration reported from Ali Haider Road, Mathpara, (Ward No. 17), Barrackpur Municipality, District North 24 Parganas. District RRT investigated the outbreak. House to house survey done. Probable cause of outbreak was contaminated water. Temporary medical camp was organized in the locality. Cases treated symptomatically. Out of 11 stool samples collected and sent to NICED Kolkata, 5 samples were positive for *V. cholerae*. Alternate supply of drinking water provided in the community. Chlorination of water sources done. ORS packets distributed. IEC done regarding safe drinking water. |
| 22 | 2013 | Maharashtra | Sindhudurg | Cholera | 74 | 0 | 5/21/2013 |  | Under control | Cases of loose motion and vomiting reported from Village Vilawade, PHC Talkat, Block Sawantwadi, District Sindhudurg. District RRT investigated the outbreak. House to house survey done. Cases treated locally. Out of 12 stool samples collected, 4 samples were positive for cholera by culture. Chlorine tablets and ORS packets distributed in the community. Chlorination of water sources done. Health education given regarding safe drinking water. |
| 23 | 2013 | Gujarat | Ahmedabad | Cholera | 51 | 0 | 6/3/2013 | 6/3/2013 | Under control | Outbreak of gastroenteritis reported from urban areas Gayatrinagar, Bachubhaino Kuvo, Lamba ward (south Zone), UHC Lamba, Ahmedabad city, District Ahmedabad. District RRT investigated the outbreak. House to house survey done. Cases treated locally by mobile medical team. Out of 39 stool samples tested at L.G. Hospital Ahmedabad, 5 samples were positive for *V. cholerae*. Pipeline leakages were observed in the community. Pipeline leakages repaired. Chlorine tablets distributed. Alternate supply of drinking water provided in the community. Health education given regarding safe drinking water, hygiene and sanitation. |
| 23 | 2013 | Karnataka | Chitradurga | Cholera | 17 | 0 | 6/5/2013 | 6/9/2013 | Under control | Cases reported from Village Bantagavi AK Hatti, SC Attimage, PHC Madadakere, PHC/CHC Hosadurga, District Chitradurga. District RRT investigated the outbreak. House to house survey done. Cases treated locally. ORS packets given to cases. 1 stool sample collected which tested positive for cholera by culture. Chlorination of water sources done by Gram Panchayat. Health education given regarding safe drinking water. |
| 23 | 2013 | Karnataka | Chitradurga | Cholera | 33 | 0 | 6/1/2013 | 6/8/2013 | Under control | Outbreak of loose motion and vomiting reported from Village Bandlarhatti, SC Talvtti, PHC Aimangla, PHC/Block Hiriyur, District Chitradurga. District RRT investigated the outbreak. House to house survey done. Cases treated locally. Out of 2 stool samples collected, 1 sample was positive for cholera by culture. Chlorination of water sources done. Health education given regarding safe drinking water. |
| 25 | 2013 | Gujarat | Gandhinagar | Cholera | 17 | 0 | 6/20/2013 | 6/20/2013 | Under surveillance | Outbreak of loose motion, vomiting and abdominal pain reported from urban area of Meena Bajar, Sachivalaya, Sec 10, UHC Palaj, Municipal Corporation of District Gandhinagar. District RRT investigated the outbreak. Active search of cases done. All cases treated at Civil Hospital, Gandhinagar. Out of 3 stool samples collected 1 stool sample was positive for cholera. Super chlorination of bore well done. Chlorine tablets, ORS packets distributed in the community. IEC materials distributed. |
| 25 | 2013 | Gujarat | Gandhinagar | Cholera | 7 | 0 | 6/20/2013 | 6/20/2013 | Under surveillance | Outbreak of loose motion, vomiting and abdominal pain reported from Urban slum Indiranagar Sec 24, Municipal Corporation of District Gandhinagar. District RRT investigated the outbreak. House to house survey done. All cases treated at Civil hospital, Gandhinagar. 1 stool sample was positive for cholera. ORS packets distributed in the community. IEC done regarding use of boiled water for drinking purpose and hand washing techniques. |
| 26 | 2013 | Maharashtra | Sangli | Cholera | 9 | 0 | 6/26/2013 | 6/27/2013 | Under control | Cases reported from Samarth Prathmik Ashramshala, Bedag village and SC of PHC Aarag. District RRT investigated the outbreak and 2 water samples taken, result awaited. Out of 8 stool samples 4 tested positive for Cholera at DPHL. It was observed that, water supply at ashram is from a bore well which was suspected to have been contaminated with fecal matter/sewage due to rains. Treatment provided to all cases and staff advised about chlorination. Health Education done. |
| 26 | 2013 | Tamil Nadu | Vellore | Cholera | 27 | 0 | 6/19/2013 | 6/24/2013 | Under control | Cases reported from Village A Kattupadi, PHC Nelvoy, Block Kaniyamabadi. Survey was initiated to trace more cases. All cases were treated. Poor personal and environmental sanitation was present. Outbreak could have occurred due to unhygienic food storage and water contamination. 3 out of 5 stool samples tested positive for *Vibrio Cholera 01 Ogawa* from Government Medical College, Vellore . |
| 26 | 2013 | West Bengal | Birbhum | Cholera | 126 | 0 | 6/25/2013 | 6/26/2013 | Under surveillance | Cases of diarrhea reported from village Kantore of SC Ghoratori under Jashpur GP of Block Dubrajpur. District & Block RRT investigated the outbreak and took 7 rectal swabs of which 6 are positive for *Vibrio Cholera 01 Ogawa*. Coliform bacteria found in water sample collected. The outbreak occurred due to use of contaminated pond water for household work. Chlorination of water done. IEC activities undertaken. |
| 27 | 2013 | Gujarat | Navsari | Cholera | 32 | 0 | 6/28/2013 | 7/2/2013 | Under control | Cases of diarrhea and vomiting reported from Ward no. 4, Maruti nagar, Ramji Park, PHC Vijalpore Urban Area, Block Jalalpore, District Navsari. District RRT investigated the outbreak. 2 out of 4 stool samples collected and sent for testing to GMC Surat were positive for Vibrio Cholerae. 4 water samples were collected and sent to Water and Sanitation Monitoring Organization (WASMO) lab, Navsari, result awaited. ORS, chlorine tablets and other medicines distributed. House to house surveillance & water chlorination done. Pipeline leakage repaired. Medical team is deployed in the area. IEC activities conducted for public awareness. |
| 27 | 2013 | Karnataka | Shimoga | Cholera | 42 | 0 | 6/17/2013 |  | Under control | Outbreak reported from Village Arebilachi, PHC, Bhadravati, District Shimoga. RRT investigated the outbreak. 15 stool samples and 13 water samples collected and sent to Medical College, Shimoga. 4 stool samples tested positive for Vibrio Cholerae (Ogawa). House to house survey done. Villagers advised on safe drinking water practices, personal hygiene and environmental sanitation. |
| 29 | 2013 | Gujarat | Anand | Cholera | 23 | 0 | 7/17/2013 | 7/18/2013 | Under control | Cases of loose motions reported from Village/SC Boria, PHC Sinhol, Block Petlad, District Anand. District RRT investigated the outbreak. House to house survey done. Pipeline leakages were observed in the community. Cases treated at local health centre. 2 stool samples collected which tested positive for cholera (O1 Ogawa). ORS packets and Chlorine tablets distributed in the community. Health education given regarding safe drinking water. |
| 29 | 2013 | Maharashtra | Dhule | Cholera | 38 | 0 | 7/15/2013 | 7/17/2013 | Under control | Outbreak reported from Village Kasturabai Aadivasi Primary & secondary Ashramshala, SC Sukwad, PHC Nardana, Block Shinkheda, District Dhule. District RRT investigated the outbreak. Affected cases were students who consumed water from bore well. Cases treated at local health centre. All 6 stool samples collected, tested positive for *V. cholerae*. 3 water samples collected were non potable. Inspection for pipeline leakages done. Chlorination of water sources done. Health education given regarding safe drinking water, hygiene and sanitation. |
| 29 | 2013 | Maharashtra | Jalgaon | Cholera | 67 | 1 | 7/10/2013 | 7/19/2013 | Under surveillance | Outbreak of gastroenteritis reported from Village Dhabe, SC Mhasave, PHC Shelave, Block Parola, District Jalgaon. District RRT investigated the outbreak. House to house survey done. People consumed water from well and hand pump. Temporary medical camp organized. Cases treated symptomatically. Out of 7 stool samples collected and sent to GMC Aurangabad, 1 sample was positive for *V. cholerae* & 1 sample for *Shigella*. Out of 6 water samples collected, 5 samples were non potable. 1 death occurred in a 10 year old female patient. Chlorination of water sources done. Instruction given to gram panchayat about regular chlorination of wells. Health education regarding safe drinking water. |
| 29 | 2013 | Tamil Nadu | Tiruchirapalli | Cholera | 13 | 0 | 7/17/2013 | 7/18/2013 | Under control | Outbreak reported from Village Thiranipalayam, SC Ootathur, PHC Alunthalaippur, Block Trichy Corporation, District Tiruchirappalli. District RRT investigated the outbreak. All 3 stool samples collected and sent to KAPV medical college were positive for cholera by culture. Chlorination of water sources done. Health education given regarding safe drinking water, hygiene and sanitation. |
| 30 | 2013 | Karnataka | Davangere | Cholera | 36 | 0 | 7/19/2013 | 7/22/2013 | Under control | Outbreak of gastroenteritis reported from Village Byaranaykanahalli, PHC K D Pura, Block Jagaluru, District Davangare. District RRT investigated the outbreak. House to house survey done. Out of 8 stool samples collected, 4 samples were positive for cholera by culture. Out of 8 water samples collected 5 water samples were non potable. Pipeline leakages were repaired. Alternate source of drinking water supplied. Chlorination of water sources done. Halogen tablets distributed in the community. Health education given. |
| 30 | 2013 | West Bengal | Purulia | Cholera | 21 | 0 | 7/26/2013 | 7/28/2013 | Under control | Cases of loose motion and vomiting reported from Village Rajra SC Murlu, Block Kashipur, District Purulia. District RRT investigated the outbreak. Active search of cases done. Cases treated symptomatically. Out of 3 stool samples collected 2 samples were positive for cholera by culture. ORS packets distributed in the community. Disinfection of the surroundings done. IEC done. |
| 30 | 2013 | Delhi | South west District | Cholera | 50 | 1 | 7/10/2013 |  | Under control | Cases of acute gastroenteritis reported from Rjokari Pahari area of South West District. State/District RRT investigated the outbreak. House to house survey done. People consumed water supplied by Delhi Jal Board. Cases treated. Of the 9 water samples collected, 6 samples were positive for faecal coliforms. 2 stool samples collected were positive for *V. cholerae* (01 ogawa)*.* Chlorine liquid and ORS packets distributed in the affected area. IEC done regarding safe drinking water. |
| 30 | 2013 | Dadra and Nagar Haveli | Dadra & Nagar Haveli | Cholera | 12 | 0 |  | 7/14/2013 | Under control | Cases were reported from Krishna Company workers Colony (block E & F), Block Samarvarni, District Dadra & Nagar Haveli. Medical team investigated the outbreak. Poor sanitary conditions were observed in the locality. Cases were treated locally. Out of 12 stool samples collected, 6 samples were positive for cholera by culture. Chlorine tablets were distributed. Health education given regarding safe drinking water, hygiene and sanitation. |
| 31 | 2013 | Assam | Kamrup M | Cholera | 14 | 0 | 8/1/2013 | 8/3/2013 | Under control | Cases of diarrhoea & vomiting reported from Sijubari, Hatigaon, Ghy, PHC Natborne, Block Capital Zone, District Kamrup Metro. District RRT investigated the outbreak. House to house survey done. Water logging was observed in the affected area. Health camp was organized in the affected area. Cases treated symptomatically. Out of 7 stool samples collected, 1 sample was positive for cholera by culture. 3 water samples collected were non potable. Chlorination of water sources done by PHED. |
| 31 | 2013 | Karnataka | Bidar | Cholera | 17 | 0 | 7/29/2013 | 8/1/2013 | Under control | Cases of loose motion and vomiting reported from Village Managalgi Nagankera, PHC Talmadgi, Block Humnabad District Bidar. District RRT investigated the outbreak. All 2 stool samples collected were positive for cholera by culture. Halogen tablets distributed in the affected area. Health education given regarding use of boiled water for drinking purposes, hand hygiene and safe food handling. |
| 31 | 2013 | Karnataka | Davangere | Cholera | 21 | 0 | 7/27/2013 | 7/29/2013 | Under control | Cases of gastroenteritis reported from Village Chikkamanahatti, PHC Palagatte, Block Jagaluru, District Davangere. District RRT investigated the outbreak. Pipe line leakages were observed in the affected area. Out of 3 stool samples collected 2 samples were positive for cholera culture. Chlorination of water sources done. Pipeline leakages were repaired. Health education given. |
| 31 | 2013 | Karnataka | Tumkur | Cholera | 34 | 0 | 7/30/2013 | 7/31/2013 | Under control | Cases of diarrhea reported from Village Gudigondanahalli gollarahatti PHC Honnavalli, Block Tiptur, District Tumkur. District RRT investigated the outbreak. 2 stool samples collected were positive for V*.Cholera (Ogawa)*. Chlorination of water sources done. Disinfection of the surroundings done. Health education given. |
| 31 | 2013 | West Bengal | Purulia | Cholera | 67 | 1 | 7/30/2013 | 7/31/2013 | Under Control | Cases of loose motion and vomiting reported from Village Radhamadhabpur, SC Monihara, Block Kashipur, District Purulia. Medical team investigated the outbreak. Open defecation and poor sanitary contiontions was observed in the community. Active case search done. Out of 2 stool samples collected, 1 stool sample was positive for V cholerae O1. All 3 water samples were non potable. IEC done regarding safe drinking water and sanitation. |
| 32 | 2013 | Karnataka | Chikkaballapur | Cholera | 24 | 0 | 8/6/2013 | 8/6/2013 | Under control | Cases of loose motion, vomiting reported from Village Nalikadaripalli, Avalabetta, PHC Mandikal, Block/District Chikkaballapur. District RRT investigated the outbreak. House to house survey done. Cases treated at District Hospital. 1 stool sample collected which tested positive for cholera by culture. Chlorination of water sources done. Health education given regarding safe drinking water, hygiene and sanitation. |
| 32 | 2013 | Maharashtra | Dhule | Cholera | 69 | 0 | 8/5/2013 | 8/8/2013 | Under control | Cases of loose motion reported from Village/SC Nimzari, PHC Wadi, Block Shirpur, District Dhule. District RRT investigated the outbreak. House to house survey done. Pipeline leakages were observed in the community. Cases treated symptomatically. Out of 12 stool samples collected, 1 sample was positive for cholera by culture. Chlorination of water sources done. Pipeline leakages were repaired. Health education given regarding safe drinking water. |
| 32 | 2013 | West Bengal | Purulia | Cholera | 13 | 0 | 7/31/2013 | 7/31/2013 | Under control | Cases of loose motion and vomiting reported from Village Raherdaga, SC Shyampaur, Block Joypur, District Purulia. District RRT investigated the outbreak. Active search of cases done. People consumed tube well water in the affected area. 1 stool sample collected was positive for cholera culture. All cases treated symptomatically. Disinfection of the surroundings done. ORS packets distributed in the community. Health talks organized in the community. IEC leaflets regarding hygiene and sanitation distributed in the community. |
| 32 | 2013 | Maharashtra | Jalgaon | Cholera | 40 | 0 | 7/29/2013 |  | Under control | Outbreak reported from Village Vishnapur Jalgaon, SC Virwade, PHC Gorgavale, Block Chopda, District Jalgaon. District RRT investigated the outbreak. House to house survey done. Clustering of cases occurred in southern part of the village. People used water from bore well. Poor sanitary conditions were observed. Temporary medical camp organized. Cases treated. Out of 5 stool samples collected and sent to GMC Aurangabad, 3 samples were positive for cholera by culture. Chlorination of water sources done. Health education given. |
| 33 | 2013 | West Bengal | Purulia | Cholera | 30 | 0 | 8/8/2013 | 8/9/2013 | Under control | Cases reported from Village Parbedia, SC Ketankeyary, Block Kashipur, Distrcit Purulia, due to consumption of contaminated water (tube well). Block Health team investigated the outbreak. Active search of case done. Open defecation and poor sanitation observed in the community. All cases treated. Out of 2 stool samples collected 1 sample was positive for cholera culture. All water samples collected were non potable. Chlorination of water sources done. ORS packets distributed. IEC leaflets regarding safe drinking water and personal hygiene. |
| 33 | 2013 | Odisha | Rayagada | Cholera | 22 | 0 | 7/27/2013 |  | Under control | Cases of loose motion and vomiting reported fro Villages Jhumurguda, Jagamunda, Lachamangunda, Nuasahi, Block Padmapur, District Rayagada. District RRT investigated the outbreak. House to house survey done. All cases treated at CHC Padamapur. Temporary health camp organized. Out of 5 stool samples collected, 4 were positive for cholera culture (01 Ogawa). Halogen tablets distributed in the community. Hand bill regarding personal hygien and sanitation was pasted on the household walls. Tube wells disinfected. Health education given regarding safe drinking water, personal hygiene. |
| 34 | 2013 | Andhra Pradesh | Medak | Cholera | 93 | 3 | 8/16/2013 | 8/25/2013 | Under surveillance | Cases of loose motion and vomiting reported from Village Ranzole, PHC Malchelma, Block Zaheerabad, District Medak. District RRT investigated the outbreak. People consumed bore well water for household activities. Open defecation observed in the community. House to house survey done. Medical camps organized in the affected area. 3 deaths occurred in patients age 7, 60 and 75 years. Out of 5 rectal swabs collected, 1 sample was positive for cholera by culture. 6 water samples collected were non potable. Chlorine tablets distributed. Disinfection of surroundings done. Health education given. |
| 34 | 2013 | West Bengal | Hooghly | Cholera | 12 | 1 | 8/21/2013 | 8/22/2013 | Under control | Cases of vomiting reported from Village Rajghat, Block Polba, District Hooghly. District RRT investigated the outbreak. Cases consumed contaminated food. All cases were workers in bakery. All 3 rectal swabs tested positive for cholera culture (Ogawa). One death occurred in 15 year old male. Health education given. |
| 34 | 2013 | West Bengal | Bankura | Cholera | 82 | 0 | 8/20/2013 | 8/21/2013 | Under control | Outbreak reported from Village Roypara, SC Jagadalla, Block Bankura-I, District Bankura. District RRT & Block health team investigated the outbreak. House to house survey done. Medicine and ORS packets given to cases. 45 cases treated at local health centre. Out of 2 stool samples collected, 1 sample was positive for cholera by culture. 7 water samples collected; result awaited. Chlorine tablets distributed. Chlorination of water sources done. Disinfection of surrounding done. IEC done regarding safe drinking water, hygiene and sanitation. |
| 34 | 2013 | West Bengal | Hooghly | Cholera | 27 | 2 | 8/22/2013 | 8/25/2013 | Under surveillance | Cases of diarrhea reported from Village/SC Gakulda, Block Pandua, District Hooghly. District RRT investigated the outbreak. Temporary medical camp organized. Cases treated symptomatically. 11 patients treated at local health centre. All 2 rectal swabs collected tested positive for Cholera culture (ogawa). 4 water samples collected; result awaited. 2 deaths occurred in patients aged 2.5 years & 12 years. Chlorine tablets and ORS packets distributed in the community. IEC done regarding safe food and drinking water, hygiene and sanitation. |
| 35 | 2013 | Delhi | New Delhi | Cholera | 50 | 1 | 8/28/2013 | 8/29/2013 | Under control | Cases of vomiting and diarrhoea reported from Indira Gandhi camp, Phase I, Loha Mandi, Naraina, District New Delhi, perhaps due to consumption of contaminated water. State/District RRT investigated the outbreak. Population of the affected area was 1500. People were of low socio economic status. Open defecation was observed in the affected jhuggi and jhopdi colony. All age groups affected. All cases treated locally. One death occurred in 45 yr old male. Out of 10 water samples collected, 3 samples were positive for cholera culture and 5 samples were positive for E. coli. All cases were advised to take treatment from Govt Dispensary. Open defecation was discouraged. ORS packets and Chlorine tablets made available at Govt Dispensary. People were advised to use boiled water for drinking purpose. IEC done. |
| 35 | 2013 | Haryana | Yamuna Nagar | Cholera | 133 | 0 | 8/28/2013 | 9/1/2013 | Under surveillance | Outbreak of loose motion reported from Village/SC Amadalpur, PHC Sabepur, Block Jagadhri, District Yamuna Nagar. District RRT investigated the outbreak. House to house survey done. Population of the affected area was 4921. Pipeline leakages were observed in the community. Temporary medical camp organized. Medicine and ORS packets given to cases. Out of 4 stool samples collected and sent to DPHL, Karnal, 1 sample was positive for cholera by culture. 10 water samples collected tested negative for residual chlorine (OT test). Further, 6 water samples collected for bacteriological testing; result awaited. Alternate supply of drinking water provided. Pipeline leakages repaired by PHED. Chlorine tablets distributed. Health talks done with local people regarding use of boiled water for drinking purpose, hygiene and sanitation. |
| 35 | 2013 | Punjab | Mohali | Cholera | 46 | 0 | 8/29/2013 | 8/31/2013 | Under control | Cases of diarrhoea reported from Dashmesh Nagar and Azadnagar Colony, Balongi, District Mohali. Medical team investigated the outbreak. House to house survey done. Out of 7 stool samples collected, 1 sample found positive for cholera culture. ORS packets and chlorine tablets distributed. Health education given regarding use of boiled water for drinking purposes hygiene and sanitation. |
| 35 | 2013 | Karnataka | Koppal | Cholera | 31 | 0 | 8/11/2013 |  | Under control | Cases of gastroenteritis reported from Village Komalapur, PHC Bannikoppa, Hirebommanal, Block Yelburgi, District Koppal, due to consumption of contaminated water (pipeline leakage). District RRT investigated the outbreak. Case treated locally. Out of 3 stool samples collected, 2 samples were positive for cholera (ogawa). Out of 3 water samples collected, 2 water samples were non potable. Chlorination of water sources done. Pipeline leakages were repaired. Alternate drinking water supply provided. Halogen tablets and ORS packets distributed in the community. Health education given regarding safe drinking water and personal hygiene. |
| 36 | 2013 | Maharashtra | Gondia | Cholera | 45 | 2 | 9/4/2013 | 9/6/2013 | Under surveillance | Cases of loose motion, vomiting and abdominal pain reported from Village & SC Datora, PHC Morwahi, Block Gondia, District Gondia. District RRT investigated the outbreak. Active search for cases done. Temporary medical camps organized. 2 death occurred in 70yr female and 48 yr male. Out of 5 stool samples collected, 3 samples were positive for cholera culture. Chlorination of water sources done. Halogen tablets distributed. IEC done regarding safe drinking water, hygiene and sanitation. |
| 36 | 2013 | Punjab | Ludhiana | Cholera | 15 | 0 | 9/5/2013 | 9/5/2013 | Under control | Cases of loose motion and vomiting reported from Village Buzgar, CHC Sidhwan Bet Ludhiana, District Ludhiana. Medical team investigated the outbreak. Out of 6 stool samples collected 4 were positive for cholera culture. Alternate supply of drinking water provided. Chlorine tablets and ORS packets distributed. Health education given regarding safe drinking water and sanitation. |
| 36 | 2013 | West Bengal | Howrah | Cholera | 164 | 0 | 9/2/2013 | 9/2/2013 | Under Control | Cases of diarrhea reported from Ghusuri, Salkia in Howarh Municipality Corporation area and Bhattanagar in Bally area, Distrcit Howrah, due to consumption of contaminated water (pipeline leakage). District RRT investigated the outbreak. House to house survey done. Out of 6 water samples collected, 4 samples were positive for cholera. All 4 stool samples collected were negative for cholera culture. Halogen tablets distributed. IEC activities done regarding safe drinking water and sanitation. |
| 36 | 2013 | West Bengal | North 24 Parganas | Cholera | 92 | 0 | 9/4/2013 | 9/7/2013 | Under control | Cases of loose motion, vomiting reported from Ward No 10 & 29, South Dumdum Municipality Area, District North 24 Paraganas. Distrcit RRT investigated the outbreak. 16 water samples collected and sent to RG Kar Medical College and NICED; 1 positive for *vibrio cholera*. ORS packets and halogen tablets distributed. IEC done. |
| 38 | 2013 | Karnataka | Koppal | Cholera | 19 | 0 | 9/16/2013 | 9/16/2013 | Under control | Cases of loose motion and vomiting reported from Village Kilaratti Tanda, PHC Mudenoor, Block Kustagi, District Koppal. District RRT investigated the outbreak. Active search for cases done. Temporary medical camps organized. Symptomatic treatment given to cases. Out of 3 stool samples collected, 1 sample was positive for cholera. 1 water sample collected was not potable. ORS packets and Halogen tablets distributed. Health education given. |
| 38 | 2013 | Punjab | Gurdaspur | Cholera | 22 | 0 | 9/17/2013 | 9/20/2013 | Under control | Cases of diarrhoea was reported from Villages Kunda, Khesal, Gopalia, PHC Behrampur, District Gurdaspur. District RRT investigated the outbreak. House to house survey done. Cases treated at local health centre. Pipeline leakages observed in the community. 9 stool samples collected were positive for cholera. ORS packets and Chlorine tablets distributed. Pipeline leakages were repaired. Health education given regarding use of boiled water for drinking purposes and hygiene. |
| 38 | 2013 | West Bengal | North 24 Parganas | Cholera | 248 | 1 | 9/22/2013 | 9/22/2013 | Under control | Cases of loose motion, abdominal pain and vomiting reported from Village Arjunpur, Rajarhat Gopalpur Municipality, District 24 Parganas, due to consumption of contaminated water (pipeline leakage). District RRT investigated the outbreak. Active search for cases done. All cases treated locally. One death occurred in 5yr old male child. Out of 4 stool samples collected, 3 stool samples were positive for cholera (01 Ogawa). 3 water samples collected; result awaited. Pipeline leakage repaired. Chlorine tablets and ORS packets distributed. Health education given. |
| 38 | 2013 | Maharashtra | Gondia | Cholera | 10 | 0 | 9/7/2013 |  | Under control | Cases of diarrhoea, vomiting and abdominal pain reported from Village & SC Navegaon, PHC Dhabepawani, Block Arjunimor, District Gondia. Medical team investigated the outbreak. House to house survey done. Cases treated locally. Out of 5 stool samples collected, 1 sample was positive for Cholera. Chlorination of water sources done. IEC done regarding safe drinking water and sanitation. |
| 38 | 2013 | West Bengal | Purulia | Cholera | 17 | 0 | 9/6/2013 |  | Under control | Cases reported from Village & SC Vosko, Block Neturia, District Purulia. Block health team investigated the outbreak. Active search for cases done. Open defecation, poor sanitary conditions were observed in the community. Out of 2 stool samples collected, one stool sample was positive for cholera. ORS packets and halogen tablets distributed. Chlorination of water sources done. Health education given regarding safe drinking water and sanitation. |
| 39 | 2013 | Haryana | Kurukshetra | Cholera | 61 | 0 | 9/29/2013 | 9/29/2013 | Under control | Cases of diarrhoea reported from Anaj Mandi, CHC Ladwa District Kurukshetra. District RRT investigated the outbreak. House to house survey done. Pipeline leakages were observed in the affected area. Health camp organized. All cases treated. Out of 4 stool samples collected, 1 stool sample was positive for Cholera culture. 5 water samples collected; result awaited. Pipeline leakages were repaired by Municipal Corporation. Alternate supple of drinking water provided. ORS packets and Halogen tablets distributed. IEC activities such as health talks were done regarding use of boiled water for drinking purpose, hand hygiene and sanitation. |
| 39 | 2013 | Maharashtra | Jalgaon | Cholera | 9 | 0 | 9/11/2013 |  | Under control | Cases of diarrhoea reported from Village Parsade, PHC Savkhedasim, Block Yaval, District Jalgaon. District RRT investigated the outbreak. House to house survey done. Pipeline leakages were observed in the community. Cases treated symptomatically. Out of 9 stool samples collected, 4 samples were positive for cholera culture. Chlorination of water sources done. Alternate supply of drinking water provided in the community. Pipeline leakages were repaired. Health education given regarding safe drinking water. |
| 40 | 2013 | Haryana | Kurukshetra | Cholera | 13 | 0 | 9/29/2013 | 10/1/2013 | Under control | Cases of loose motion and vomiting reported from Village Buhawa, PHC Deeg, District Kurukshetra. District RRT investigated the outbreak. House to house survey done. All cases treated. Out of 2 stool samples collected, 1 stool sample was positive for Cholera culture. 9 water samples collected; result awaited. ORS packets and Halogen tablets distributed. IEC done regarding use of boiled water for drinking purpose, hand hygiene and sanitation. |
| 40 | 2013 | Karnataka | Bellary | Cholera | 20 | 0 | 9/28/2013 | 10/1/2013 | Under control | Cases of loose motion and vomiting reported from Village Metri, PHC Devasamudra, Block Hospet, District Bellary. District RRT investigated the outbreak. House to house survey done. Pipeline leakages were observed in the community. All cases treated. 1 sample collected tested positive for cholera culture. Out of 4 water sample collected, 2 samples were non potable. Halogen tablets distributed in the community. Pipeline leakages were repaired. Health education given regarding use of boiled water for drinking purpose and sanitation. |
| 40 | 2013 | Chhattisgarh | Narayanpur | Cholera | 66 | 19 | 9/10/2013 |  | Under surveillance | Outbreak of diarrhoea reported from Villages Binaginda Orchha, Jharwar Orchha, Chhindpur Orchha, Konge Orchha and Kodonar SC Pangud and Konge, PHC Garpa, District Narayanpur. Medical team investigated the outbreak. A total of 66 cases and 19 deaths were reported in the following villages; Binaginda Orchha (42 cases, 8 deaths), Jharwar Orchha (11 cases, 5 deaths), Chhindpur Orchha (5 cases, 5 deaths), Konge Orchha (5 cases) and Kodonar (3 cases, 1 deaths). Temporary health camps organized. All cases treated. Out of 4 stool samples collected, 1 sample was positive for V.cholera (Hikojima). Chlorination of water sources done. IEC done. |
| 40 | 2013 | West Bengal | Bankura | Cholera | 39 | 0 | 9/19/2013 |  | Under control | Cases of loose motions and vomiting reported from Village Bindna, SC Kharbona, Block Chhatna, and District Bankura. District RRT and Block health team investigated the outbreak. Active search for cases done. All cases treated locally. All 3 rectal swabs collected were positive for Cholera (01 Ogawa). Chlorination of water sources done. Chlorine tablets and ORS packets distributed. Health education given regarding safe drinking water and sanitation. |
| 41 | 2013 | Assam | Dibrugarh | Cholera | 11 | 0 | 10/8/2013 | 10/12/2013 | Under control | Cases of diarrhoea reported from Village Tamulbari T.E., SC Jilliguri, Block Lahowal, District Dibrugarh. District RRT investigated the outbreak. House to house survey done. Cases treated locally. 2 stool samples collected were positive for cholera (01 Ogawa). 2 water samples collected; result awaited. Chlorination of water sources done. Health education given regarding safe drinking water and sanitation. |
| 41 | 2013 | Haryana | Ambala | Cholera | 60 | 1 | 10/9/2013 | 10/13/2013 | Under surveillance | Cases of diarrhoea reported from Village Toba, PHC Saha, Block Mulana, District Ambala. Medical team investigated the outbreak. House to house survey done. Pipeline leakages were observed in the affected area. Health camp organized. All cases treated. Out of 11 stool samples collected, 2 stool samples were positive for Cholera culture. 11 water samples collected; result awaited. Pipeline leakages were repaired. Alternate supply of drinking water provided. ORS packets and Halogen tablets distributed. IEC done regarding use of boiled water for drinking purpose and sanitation. |
| 41 | 2013 | Assam | Dibrugarh | Cholera | 17 | 0 | 9/28/2013 |  | Under control | Cases of diarrhoea reported from Village Nandanbon T.E., Block Tegakhat, District Dibrugarh. District RRT and Block health team investigated the outbreak. Active search for cases done. People consumed water from tube well. Poor hygiene and sanitation observed in the community. Cases treated at local health center. 2 stool samples collected were positive for cholera culture. Chlorination of water sources done. IEC done regarding safe drinking water and personal hygiene. |
| 41 | 2013 | West Bengal | North 24 Parganas | Cholera | 698 | 0 | 10/9/2013 |  | Under control | Cases of loose motion and vomiting reported from urban areas of Pratapgarh, Barishalnagar, Uttar Pratapgarh, Ward no 11 in North Dum Dum Municipality, Subdivision Barrackpore, District North 24 Parganas. Medical team investigated the outbreak. Active search for cases done. Temporary medical camps organized. All cases tread symptomatically. Out of 6 rectal swabs collected 3 samples were positive for cholera culture. Out of 4 water samples collected 1 sample was positive for cholera culture. Chlorination of water sources done. Halogen tablets and ORS packets distributed in the community. IEC done. (*instead of 10th September 2013 mentioned in 41st week report (week ending 13th October 2013), the confirmed date of start of outbreak is 9th October 2013) |
| 42 | 2013 | Maharashtra | Kolhapur | Cholera | 13 | 1 | 10/11/2013 | 10/15/2013 | Under contro | Cases of diarrhoea reported from Village AP Bhatiwade, SC Hedawade, PHC Minche Kh, Block Bhudargad, District Kolhapur. District RRT investigated the outbreak. House to house survey done. Pipeline leakages were observed in the community. Cases treated at local health centre. 1 stool sample was positive for Cholera. Chlorination of water sources done. Chlorine tablets distributed in the community. Pipeline leakages repaired. Health education given regarding safe drinking water, hygiene and sanitation. |
| 42 | 2013 | West Bengal | Purulia | Cholera | 70 | 0 | 10/14/2013 | 10/15/2013 | Under control | Cases of gastroenteritis reported from Village Misirdih, SC Patuara, Block Arsha, District Purulia. District RRT and Block health team investigated the outbreak. Cases treated symptomatically. 2 stool samples collected were positive for Vibrio cholerae (O1 Ogawa). 2 water samples were non potable. Chlorine tablets and ORS packets distributed in the community. IEC done. |
| 43 | 2013 | Karnataka | Mandya | Cholera | 226 | 0 | 10/19/2013 | 10/22/2013 | Under control | Cases of diarrhoea reported from Village/SC Holalu, Block/District Mandya. District RRT investigated the outbreak. House to house survey done. Temporary medical camps organized in the community. Cases treated symptomatically. All 13 stool samples tested positive for *vibrio cholera* on culture. Out of 8 water samples tested by H2S test, 6 found not potable. Health education given regarding safe drinking water and sanitation. |
| 43 | 2013 | Maharashtra | Thane | Cholera | 59 | 1 | 10/17/2013 | 10/23/2013 | Under Surveillance | Cases of diarrhea and vomiting reported from Dabhon Ashramshala, SC Rankol, PHC Aina, Taluka Dahanu, District Thane. On investigation by District RRT it was observed that water consumed by the community was contaminated and irregularly chlorinated. 4 Water samples found non potable and out of 15 stool samples sent to DPHL Thane 6 stool samples tested positive for *vibrio cholerae 01 Ogawa*. Death of 14 year old boy occurred due to severe dehydration. Referred cases were treated at SDH Dahanu and Kasa. Medical examination of all students and staff done. Water supply from contaminated source stopped and alternate water supply provided. |
| 43 | 2013 | West Bengal | Purulia | Cholera | 23 | 0 | 10/23/2013 | 10/23/2013 | Under Surveillance | Cases of loose motions reported from Village Ukada, S/C Kornag, Block Bagmundi, District Purulia. District RRT investigated the outbreak. Out of 2 stool samples collected, one shows growth of *vibrio cholerae 01* and 2 water samples found contaminated with coliforms. Outbreak was attributed to consumption of contaminated water and poor hygienic habits. Active search of cases and line listing done. Disinfection of drinking water done. |
| 43 | 2013 | Maharashtra | Pune | Cholera | 8 | 0 | 9/25/2013 |  | Under Control | Cases of diarrhea reported from village & SC Sarola, PHC Bhongawali, Block Bhor, District Pune. On investigation contamination of well water and pipeline leakages observed. Out of 10 water samples tested 6 not potable and 2 out of 4 stool samples tested found positive for *vibrio cholerae*, Active surveillance and repair of pipelines done. Medichlor and treatment provided. Cleaning of water sources done. |
| 44 | 2013 | Odisha | Cuttack | Cholera | 23 | 1 | 10/27/2013 | 10/28/2013 | Under control | Outbreak of diarrhoea reported from Ward no. 46, Jagannath colony, Cuttack Municipal Corporation Khannagar, District Cuttack. Medical team investigated the outbreak. Active search for cases done. All cases treated locally. Deceased is 17 year old female. Out of 3 rectal swabs collected, 2 were positive for cholera (*01* Ogawa). Halogen tablets and ORS packets were distributed CMC HW. Health education given. |
| 44 | 2013 | West Bengal | Hooghly | Cholera | 52 | 0 | 10/27/2013 | 10/28/2013 | Under control | Cases of loose motion and vomiting reported from Village Natungram, SC Barochowka, Block Mogra, District Hooghly. District RRT investigated the outbreak. 2 rectal swab samples were positive for cholera (O1 Ogawa). ORS packets were distributed along with chlorine tablets. People were advised to practice good hygienic practices. IEC activities were undertaken regarding safe drinking water. |
| 44 | 2013 | West Bengal | Bankura | Cholera | 86 | 0 | 10/24/2013 | 10/28/2013 | Under control | 22 Cases of loose motion and vomiting reported from Village Sonatapal, SC Baliara, Block Onda, District Bankura. District RRT and Block health team investigated the outbreak. Active search for cases done. All cases treated at local health centre. 1 rectal swab sample and 2 water samples collected; result awaited. Halogen tablets and ORS packets were distributed. Chlorination of water sources done. Health education was given regarding safe drinking water and personal hygiene.46 cases occurred in week 45th (week ending 10th November 2013). Out of 8 samples tested, 3 samples were positive for cholera (01 Ogawa). |
| 44 | 2013 | West Bengal | Bankura | Cholera | 110 | 0 | 10/25/2013 | 10/28/2013 | Under control | Cases of diarrhoea reported from Village Sihar, Block Kotulpur, District Bankura. District RRT investigated the outbreak. House to house survey done. Symptomatic treatment given to cases. 2 rectal swabs collected were positive for cholera (Ogawa). 4 water samples from tube wells and pond were collected; result awaited. Halogen tablets and ORS packets were distributed. Health education given regarding safe drinking water and personal hygiene.Further 60 cases occurred in week 45th (week ending 10th November 2013).Out of 8 samples tested, 1 sample tested positive for cholera (01 Ogawa). |
| 44 | 2013 | West Bengal | Bankura | Cholera | 86 | 0 | 10/25/2013 | 10/28/2013 | Under control | Cases of gastroentritis reported from Village Basaura, SC Mankhamar, Block Onda, Distt Bankura. District RRT investigated the outbreak. House to house survey done. All cases treated symptomatically. Out of 8 rectal swabs collected, 3 samples were positive for cholera (01Ogawa). 13 water samples from tube wells and pond were collected; result awaited. Halogen tablets and ORS packets were distributed. Health education given regarding safe drinking water and personal hygiene. |
| 44 | 2013 | Karnataka | Gulbarga | Cholera | 20 | 0 | 10/15/2013 |  | Under control | Cases of gastroenteritis reported from Villages Jawalga B, PHC Gola B, Block Aland, District Gulbarga. District RRT investigated the outbreak. Active search for cases done. Cases treated symptomatically. Out of 5 stool samples collected, 1 stool sample was positive for cholera culture. Out of 14 water samples collected, 8 water samples were non potable. Chlorine tablets and ORS packets distributed. Chlorination of water sources done. Health education given regarding safe drinking water and sanitation. |
| 44 | 2013 | West Bengal | Dakshin Dinajpur | Cholera | 118 | 1 | 10/19/2013 |  | Under surveillance | Cases of loose motions and vomiting reported from Village & SC Gopalbati, Block Balurghat, District Dakshin Dinajpur. District RRT and Block health team investigated the outbreak. House to house survey done. One death occurred in 35 yr old male. Symptomatic treatment given to cases. One stool sample collected was positive for cholera by culture. Health education given. |
| 45 | 2013 | Assam | Dibrugarh | Cholera | 18 | 0 | 10/30/2013 | 11/4/2013 | Under Control | Cases reported from Village Lengrai TE, Block Tengakhat, District Dibrugarh. Block Health Team investigated the outbreak. Active search for cases done. All cases treated at local health center. 2 rectal swabs collected were positive for cholera (01 Ogawa). Out of 9 water samples collected 4 samples were non potable. Halogen tablets were distributed. IEC done regarding for safe drinking water and sanitation. |
| 45 | 2013 | Assam | Tinsukia | Cholera | 11 | 1 | 11/4/2013 | 11/7/2013 | Under Control | Cases of loose motion reported from Village Deohal TE, PHC Hapjan, District Tinsukia. Block Health Team investigated the outbreak. House to house survey done. Temporary medical camps organized. All cases treated symptomatically. 2 rectal swabs collected were positive for cholera (01 Ogawa). Halogen tablets and ORS packets were distributed. IEC done regarding for safe drinking water and sanitation. |
| 45 | 2013 | Andhra Pradesh | Mahabubnagar | Cholera | 62 | 0 | 11/5/2013 | 11/7/2013 | Under surveillance | Cases of diarrhoea reported from Village Medipally, SC Motlampally, PHC Thippadampally, Block Athmakur, District Mahbubnagar. District RRT investigated the outbreak. Pipeline leakages were observed in the community. House to house survey done. Medical camp organized. Cases treated. Out of 8 stool samples collected 4 samples were positive for cholera. 7 water samples collected; result awaited. Chlorination of water sources done. Pipeline leakages repaired. IEC done regarding personal hygiene and sanitation. |
| 47 | 2013 | Karnataka | Bellary | Cholera | 18 | 0 | 11/21/2013 | 11/23/2013 | Under control | Cases of gastroenteritis reported from Village/PHC Hacholi, Block Siruguppa, District Bellary. District RRT investigated the outbreak. House to house survey done. Pipeline leakages were observed in the community. Cases treated symptomatically. Out of 4 stool samples collected 2 samples were positive for cholera culture. 3 water samples collected, result awaited. Pipeline leakages were repaired. Halogen tablets distributed. IEC done regarding safe drinking water and personal hygiene. |
| 48 | 2013 | West Bengal | Hooghly | Cholera | 13 | 0 | 11/27/2013 | 11/28/2013 | Under control | Cases of diarrhoea reported from Village/SC Bhadua and Santrapara, Block Chanditala I, District Hooghly. Block health team investigated the outbreak. House to house survey done. All cases treated locally. Out of 5 rectal swabs collected 3 samples were positive for cholera culture (O1 Ogawa). 2 water samples collected; result awaited. ORS packets and chlorine tablets distributed in the area. IEC regarding safe drinking water and personal hygiene done. |
| 48 | 2013 | West Bengal | Purulia | Cholera | 43 | 0 | 11/26/2013 | 11/27/2013 | Under control | Cases of loose motion reported from Village / SC Kachahatu, Block Kotshila, District Purulia. Medical team investigated the outbreak. House to house survey done. All cases treated symptomatically. Out of 2 rectal swabs collected 1 sample was positive for cholera culture (O1 Ogawa). ORS packets and halogen tablets distributed in the community. Health education given regarding safe drinking water and sanitation. |
| 50 | 2013 | Maharashtra | Sangli | Cholera | 99 | 1 | 12/7/2013 | 12/12/2013 | Under control | Cases of loose motion and vomiting reported from Village Ozarde, SC Surul, PHC Peth, Block Walwa, District Sangli. Medical team investigated the outbreak. House to house survey done. Pipeline leakages were observed in the affected area. Symptomatic treatment given to cases. All 7 stool samples collected were positive for cholera culture. Chlorination of water sources done. Pipeline leakages were repaired. Health education given regarding safe drinking water and sanitation. |
| 51 | 2013 | Tamil Nadu | Krishnagiri | Cholera | 19 | 0 | 12/15/2013 | 12/19/2013 | Under control | Cases of diarrhoea were reported from Village Mathagondapalli, SC Mathagondapalli, PHC Krishnagiri, Block Thally, District Krishnagiri. District RRT investigated the outbreak. House to house survey done. All cases treated at local health centre. Pipeline leakages were observed in the community. Out of 5 stool samples collected, 2 stool samples were positive for cholera culture. 2 water samples collected were non potable. Chlorination to water sources done. IEC done regarding safe water drinking and personal hygiene. |
|  |  |  |  |  |  |  |  |  |  |  |
| Wk No. | Year | State | District | Disease | Cases | Deaths | Date of Outbreak | Date of Reporting | Current Status | Comments |
| 2 | 2014 | Odisha | Ganjam | Cholera | 42 | 0 | 1/1/2014 | 1/12/2014 | Under control | Cases of diarrhoea reported from Village/SC/PHC Padmapur, CHC Badagada, Block Soruda, District Ganjam. Block Health team investigated the outbreak. Active search for cases done. Out of 5 rectal swabs collected, 1 sample was positive for cholera culture. Cases treated locally. Disinfection of surrounding done. Chlorine tablets distributed. IEC activities regarding personal hygiene and safe drinking water done. |
| 3 | 2014 | Odisha | Kendrapara | Cholera | 94 | 0 | 1/14/2014 | 1/15/2014 | Under control | Outbreak reported from Villages Khamasahi, Dangamala, Nuagaon & Nalitapatia, SC & PHC Dangamala, Block Rajnagar, District Kendrapara. District RRT and Block Health team investigated the outbreak. Cases had consumed food in a birthday ceremony. Cases presented with vomiting, loose motion. Out of 5 rectal swabs collected, 1 sample was positive fro cholera culture (O1 Ogawa). 3 water samples collected; result awaited. All cases treated symptomatically. Health education given regarding food hygiene and sanitation. |
| 3 | 2014 | Odisha | Ganjam | Cholera | 41 | 0 | 1/2/2014 |  | Under control | Cases of diarrhoea reported from Village/SC/Block Patrapur, District Ganjam. Medical team investigated the outbreak. House to house survey done. Poor sanitary conditions and open defecation were observed in the community. Out of 8 rectal swabs collected 1 sample was positive for cholera by culture (O1 Ogawa). 3 water samples were collected; result awaited. Medical camps organized in the community. All cases treated symptomatically. Chlorine tablets distributed. IEC done regarding safe drinking water, personal hygiene and sanitation. |
| 4 | 2014 | West Bengal | Malda | Cholera | 69 | 0 | 1/22/2014 | 1/23/2014 | Under control | Cases of loose motion and vomiting reported from Ward No. 17 Rasiladah Colony, Old Malda Municipality, District Malda. Medical team investigated the outbreak. House to house survey done. Poor sanitary conditions were observed in the locality. Medical camps organized. Cases treated symptomatically. 3 stool samples and 4 water samples collected; one was positive for Cholera. Chlorination of water sources done. Disinfection of surrounding done. ORS packets and chlorine tablets distributed. IEC done regarding safe drinking water, hygiene and sanitation. |
| 14 | 2014 | West Bengal | Birbhum | Cholera | 31 | 0 | 4/3/2014 | 4/4/2014 | Under Control | Cases of loose motion and vomiting reported from Village Bhabanipur, SC Chinpai, Block Dubrajpur, District Birbhum. District RRT investigated the outbreak. House to house survey done. People consumed pond water for daily activities. All cases treated symptomatically. 2 rectal swabs sent; result awaited. ORS packets distributed. Disinfection of pond and well done. IEC done regarding safe drinking water and sanitation. |
| 20 | 2014 | Karnataka | Mysore | Cholera | 32 | 0 | 5/15/2014 | 5/18/2014 | Under Control | Cases of diarrhoea reported from Village/SC KK Thanda, PHC MM Wada, Block Hadagalli, District Mysore. District RRT investigated the outbreak. Pipeline leakages were observed in the community. Out of 05 water samples tested 4 samples were non potable. Out of 7 stool samples tested at Mysore Medical College, 03 were positive for Cholera by culture. Chlorine tablets distributed. IEC activities regarding personal hygiene and safe drinking water done. |
| 22 | 2014 | Karnataka | Davangere | Cholera | 98 | 2 | 5/18/2014 |  | Under Control | Cases of diarrhea reported from village Yarala Bannikoodu, PHC/CHC Kulambi, Taluk Honnali, District Davangere. District RRT investigated the outbreak. A total of 12 stool samples collected and sent to JJMC Davangere, 03 tested positive for V Cholera by Culture. A total of 27 water samples collected and sent for testing, 18 water samples are not potable, and result of 7 samples awaited. Water supply from contaminated source stopped. Chlorination of well done and halogen tablets distributed. House to house survey done and serious cases were referred. Health education given to people. |
| 23 | 2014 | Haryana | Gurgaon | Cholera | 60 | 0 | 6/9/2014 | 6/9/2014 | Under surveillance | Cases of diarrohea reported from Carterpuri, PHC / Distt Gurgaon. It was observed that water supply was contaminated and people were living in poor environmental conditions. A total of 05 stool samples were taken and sent to NCDC Delhi out of which 03 were positive for Vibrio Cholera O1 by Culture. 2 water samples were also taken for bacteriological test; result awaited. OT test was done on 20 water samples and all found unfit for drinking. IEC done in the area. ORS packets and chlorine tablets are distributed. |
| 23 | 2014 | Karnataka | Chitradurga | Cholera | 25 | 0 | 6/5/2014 | 6/7/2014 | Under control | Cases of diarrhea reported from Village Ramajogihalli, PHC / SC Yarabahalli, Taluk Hiriyur, District Chitradurga. RRT investigated the outbreak. 10 stool sample & 3 water samples (H2S test ) were collected; 2 stool sample positive for Cholera by Culture; result awaited for water samples. There was contamination of water due to pipeline leakage. Gram Panchayat instructed to arrange alternate source of water supply. Cases managed in temporary clinic. ORS distributed. Health education given. |
| 30 | 2014 | Punjab | Ludhiana | Cholera | 63 | 3 | 7/22/2014 | 7/25/2014 | Under surveillance | Cases of diarrhea reported from Parkash Nagar, Jawaddi, District Ludhiana. Cases occurred due to consumption of contaminated water. A total of 06 water and 05 stool samples were collected and sent to CMC Ludhiana; 03stool samples tested positive for V Cholera Ogawa. Medical camp organized in the area. |
| 30 | 2014 | Punjab | Patiala | Cholera | 28 | 0 | 7/27/2014 | 7/27/2014 | Under control | Cases of diarrhea reported from Varaichan Patti, Samana, District Patiala. There is mixing of drinking water with drain water in the affected area. A total of 05 water samples & 04 stool samples were collected & sent for analysis to State Public Health Lab Patiala and GMC Patiala respectively. one stool samples tested positive for V Cholera Ogawa while result of water sample is awaited. House to house survey done. Medical camp organized. ORS packets & chlorine tablets distributed. IEC activities conducted. |
| 30 | 2014 | Punjab | Hoshiarpur | Cholera | 86 | 0 | 7/20/2014 | 7/21/2014 | Under surveillance | Outbreak of cholera reported from Sundernagar, District Hoshiarpur. There was mixing of sewage water with potable water due to leakage in potable water pipeline. Medical camp organized in the affected area. 5 water samples collected and sent to State Public Health Lab Chandigarh; result awaited. 22 stool samples collected and sent to DPL Hoshiarpur, 14 stool samples were positive for Vibrio Cholera Ogawa strain. Chlorine tabs and ORS packets were distributed. Health education given. |
| 30 | 2014 | Chhattisgarh | Mahasamund | Cholera | 46 | 1 | 7/12/2014 |  | Under surveillance | Cases reported from Block Basna, CHC Basna, PHC Chanat, SC Ajgarkhar, Village Ajgarkhar, District Mahasamund. Cases presented with diarrhea and gastroenteritis. A total of 04 samples taken for cholera confirmation and sent to JNM Medical college, all samples positive for Cholera O1 Ogawa. Water sources were chlorinated and health education was given. |
| 30 | 2014 | Odisha | Khordha | Cholera | 10 | 0 | 7/10/2014 |  | Under control | Cases of Diarrhoea reported from Village Krushnapur, SC Golabal, CHC Haladia, Block Khordha, GP Orabarsingh, District Khordha. District RRT investigated the outbreak. Active case search done. 3 water samples and 3 rectal swabs sent to laboratory; result awaited. Disinfection of water sources done. Halogen tablets distributed. Symptomatic treatment given. IEC done. |
| 31 | 2014 | West Bengal | Hooghly | Cholera | 56 | 0 | 8/3/2014 | 8/3/2014 | Under Surveillance | Cases of loose watery stool reported from Kamchey, BPHC Goghat - I, District Hooghly. District RRT investigated the outbreak. Active search for cases done. All cases treated symptomatically. Out of 03 rectal swabs 01 sample positive for Vibrio cholera 01 Ogawa. 01 water sample sent; result awaited. Chlorination of water sources done. ORS packets distributed. Health education given regarding food hygiene and sanitation. |
| 32 | 2014 | West Bengal | North 24 Parganas | Cholera | 445 | 0 | 7/18/2014 |  | Under control | Outbreak reported from Panihati Municipal Area, District North 24 Parganas. 3 rectal swabs were collected & sent to RG Kar MCH out of which 2 were found positive for Vibrio Cholerae. 2 water samples were positive for E Coli. ORS packets, Halogen tablets distributed. Medical camp was organized. All cases treated. |
| 33 | 2014 | Gujarat | Sabarkantha | Cholera | 23 | 1 | 8/12/2014 | 8/13/2014 | Under Surveillance | Cases of diarrhea reported from Village /SC Sabalpur, PHC Bolundra, Block Modasa, District Sabarkantha. District RRT investigated the outbreak. Active search for cases done. Probable cause of outbreak was contamination of drinking water due to pipeline leakage. 02 water sample sent to Microbiology Department, Himmatnagar Hospital; result awaited. Out of 10 stool sample tested 06 were positive for cholera. Chlorine tablets & ORS packets distributed. IEC done regarding personal hygiene and safe drinking water. |
| 33 | 2014 | Odisha | Kendrapara | Cholera | 13 | 0 | 8/9/2014 | 8/17/2014 | Under Control | Cases of diarrhoea reported from Village Chandanpur, SC Ostapur, PHC Ayeba,CHC Indupur, District Kendrapara. District & Block RRT investigated the outbreak. Active search for new cases done. Probable cause of outbreak was contamination of drinking water. All cases treated symptomatically. Out of 02 water samples tested 01 positive for Vibrio cholera NAD and 01 positive for E.Coli. 02 rectal swabs sent to SCBMC, Cuttack, positive for Vibrio cholera. IEC done regarding personal hygiene and safe drinking water. |
| 33 | 2014 | Punjab | Ludhiana | Cholera | 10 | 0 | 8/16/2014 | 8/17/2014 | Under Control | Cases of diarrhoea reported from Labour Colony, Omax Construction Company, Thakkarwal, District Ludhiana. District RRT investigated the outbreak. Active search for new cases done. Cases consumed common food in a social ceremony. All cases treated symptomatically. 05 water samples sent to state public health Lab, Mohali; result awaited. Out of 03 stool samples sent to CMC, Ludhiana 02 samples showed growth of Vibrio cholera Ogawa. Health education given regarding food hygiene and sanitation. |
| 34 | 2014 | Karnataka | Chitradurga | Cholera | 38 | 0 | 8/22/2014 | 8/23/2014 | Under Control | Cases of loose motion and vomiting reported from Village Vaddarahatti, PHC Yalandur, Block CN Halli, District Tumkur. District RRT investigated the outbreak. Active search for cases done. Pipeline leakages were observed in the community. Cases treated symptomatically. Temporary medical camp organized. Out of 9 stool samples collected 1 sample was positive for cholera culture. 4 water samples collected; result awaited. Alternate supply of drinking water provided. Chlorination of waters sources done. ORS packets distributed. Health education given regarding safe drinking water and sanitation. |
| 34 | 2014 | West Bengal | Purulia | Cholera | 67 | 0 | 8/18/2014 | 8/18/2014 | Under Surveillance | Cases reported from Village Fosko, SC Rangamati, Block Arsha and District Purulia. District RRT investigated the outbreak. Active search for cases done. Probable cause of outbreak was contamination of drinking water due to pipeline leakages. Open defecation and poor hygiene observed in the community. All cases treated symptomatically. Out of 3 stool samples sent 01sample tested positive for Vibrio cholerae 01 Ogawa. Out of 3 water samples tested 02 samples found non potable. Chlorination of water sources done. Disinfection of the surrounding done. ORS packets distributed in the community. Health education given regarding safe drinking water and personal hygiene. |
| 35 | 2014 | Haryana | Panchkula | Cholera | 761 | 0 | 8/29/2014 | 8/30/2014 | Under Control | Cases of diarrhoea reported from Sector 19, Panchkula. Probable cause of outbreak is contamination of drinking water. A total of 31 stool samples, 60 water samples collected; 07 positive for Vibrio Cholera, 24 water samples fit for consumption while result of 36 water samples awaited. OT test was done on 245 water samples; of all samples 07 found unfit.. RRT visited the affected area. House to house survey is going on. IEC done regarding hygienic practices and sanitation measures. Chlorine tablets were distributed for household chlorination. |
| 35 | 2014 | Odisha | Cuttack | Cholera | 14 | 0 | 8/29/2014 | 8/29/2014 | Under Surveillance | Cases of diarrhoea reported from Village Sardar Kharida, SC Bada Samantarapur, PHC Mangarajpur, CHC Tangi, District Cuttack. Cases occurred due to consumption of contaminated well water. A total of 02 stool and 02 water samples collected and sent to State Referral Lab, Cuttack; stool samples were positive for Cholera and water samples tested positive for cholera and coliforms. Medical team investigated the situation. Health camp organized in the area. |
| 35 | 2014 | West Bengal | Hooghly | Cholera | 58 | 0 | 8/22/2014 | 8/25/2014 | Under Surveillance | Cases of diarrhoea reported from various areas of Tarakeswar, Gohami , GP Baligori II, Jamna,, Pandua , District Hooghly. Block RRT investigated the outbreak. Out of 6 stool samples tested at NICED, 1 was positive for Vibrio cholerae O1 Ogawa and result of 03 samples are awaited. Halogen tablets distributed. IEC done. |
| 36 | 2014 | Assam | Lakhimpur | Cholera | 59 | 1 | 9/4/2014 | 9/7/2014 | Under Surveillance | Cases of diarrhoea and vomiting reported from BPHC Boginodi, MPHC Seajuli affected area Joyhing TE and District Lakimpur. District RRT investigated the outbreak. Active search for case done. Poor sanitary condition was observed in the community. All cases treated symptomatically. Out of 08 stool samples tested 01 sample showed growth of Vibrio cholera 01 serotype (Ogawa).03 water samples tested & found to be non potable. Health education given regarding food hygiene and sanitation. |
| 37 | 2014 | Tamil Nadu | Cuddalore | Cholera | 16 | 0 | 9/11/2014 | 9/14/2014 | Under Surveillance | Cases of diarrhoea reported from Village Semmankuppam, PHC Karaikadu, Block / District Cuddalore. District RRT investigated the outbreak. Active survey done. All cases treated symptomatically.Out of 05 stool samples sent to DPHL Cuddalore 02 tested positive for Vibrio cholerae. Chlorination of water sources done. Health education done regarding safe drinking water and sanitation. |
| 39 | 2014 | Punjab | Sangrur | Cholera | 94 | 0 | 9/23/2014 | 9/23/2014 | Under Control | Cases reported from Village Lehra Gaga, District SangrurDistrict RRT visited the area. House to house survey done. All cases treated symptomatically. Probable source of infection was consumption of contaminated tap water. Out of 08 water samples collected and sent to District Public Health Lab Mohali, 04 samples were found non potable. Out of 11 stool samples collected and sent to GMC Patiala 1 tested positive for Cholera (Ogawa). Health education given regarding food & water hygiene and sanitation. |
| 40 | 2014 | Gujarat | Vadodara | Cholera | 35 | 0 | 10/1/2014 | 10/5/2014 | Under control | Cases of diarrhoea and vomiting reported from Village Por, PHC Varnama District Vadodara. District RRT investigated the outbreak. Active search for cases done. Pipeline leakages were observed.02 water sample tested found to be potable.1 stool sample collected was positive for Cholera by culture. Pipeline leakage repaired. Cholirnation of water sources done. IEC done regarding personal hygiene and safe drinking water. |
| 40 | 2014 | West Bengal | Dakshin Dinajpur | Cholera | 23 | 0 | 9/30/2014 | 10/1/2014 | Under Surveillance | Cases reported from Village Gerul, SC Uttar Belaghat, Block Bansihari, District Dakshin Dinajpur. District RRT investigated the outbreak. House to house survey done.All cases treated symptomatically. One stool sample sent to NICED, Kolkata tested positive for Vibrio cholerae. Health education done regarding safe drinking water and sanitation. |
| 40 | 2014 | West Bengal | West Medinipur | Cholera | 148 | 0 | 9/24/2014 | 9/29/2014 | Under Surveillance | Cases reported from Ward no. 7 &8 under KGP municipality Rajogram para and District Medinipur West. District RRT investigated the outbreak. House to house survey done. All cases treated locally. Probable cause of outbreak was contamination of drinking water due to pipeline leakages. 2 stool samples tested by dipstick found positive for Vibrio cholerae. 2 water samples sent to PHE lab; result awaited. 8 stool samples sent to MMCH for culture; result awaited. Pipeline leakages have been repaired by PHE and Municipality. Disinfection of surrounding done. Chlorine tablets and ORS packets distributed. Health education done regarding safe drinking water and sanitation. |
| 42 | 2014 | Odisha | Balangir | Cholera | 40 | 1 | 10/6/2014 | 10/13/2014 | Under Surveillance | Cases of diarrhoea reported from Village / GP Khalikani, CHC/ Block Saintala, District Balangir. Block RRT investigated the outbreak. House to house survey done. Probable cause of outbreak was contamination of drinking water. All 04 rectal swabs sent to Regional medical research centre, Bhuvaneshwar tested positive for Vibrio cholerae. One death occurred in a 40year old lady due to the condition. Chlorination of water sources done. Halogen tablets & ORS packets distributed. Health education imparted regarding safe drinking water and sanitation. |
| 44 | 2014 | Punjab | Moga | Cholera | 13 | 0 | 10/30/2014 | 10/30/2014 | Under Control | Cases of abdominal pain vomiting and diarrhoea reported from Danda Mandi, District Moga. District RRT investigated the outbreak .House to house survey done.Poor sanitary conditions were observed in the community. Out of 05 water samples sent to SPHL, Chandigarh 04 water samples found non potable. Out of 06 stool samples sent to CMC Ludhiana 04 samples showed growth of Vibrio cholera. Alternate source of water supply arranged. Chlorine tablets distributed. Health education given regarding safe drinking water and sanitation. |
| 45 | 2014 | Bihar | East Champaran | Cholera | 21 | 1 | 10/29/2014 | 11/6/2014 | Under Surveillance | Cases of dehydration, abdominal pain and headache reported from Village Ekdarwa, Ward No 07, PHC Ekdarwa, District East Champaran. RRT visited the area. Active survey done. All cases treated symptomatically. Out 06 stool samples collected and sent to Duncan hospital.Raxual 01 sample tested positive for cholera by hanging drop. Health education given regarding sanitation and hygiene. |
| 46 | 2014 | Gujarat | Chota Udaipur | Cholera | 94 | 1 | 11/9/2014 | 11/12/2014 | Under Surveillance | Cases of diarrhea and vomiting reported from Village Sankheda Gundhicha, Block Sankheda, District Chota Udaipur District RRT investigated the outbreak. Active case search done. All cases treated symptomatically. One 28 year old male died due to the condition.Out of 43 stool samples tested 14 sample tested positive for Cholera by culture at SSG Medical College Vadodra. 03 water samples sent to Public Health Lab Vadodra, found to be non-potable. Chlorination of water sources done. ORS packets distributed. Health education done regarding safe food & water hygiene and sanitation. |
| 46 | 2014 | Kerala | Palakkad | Cholera | 2 | 0 | 11/5/2014 | 11/13/2014 | Under Surveillance | Cases of acute watery diarroea, in Village-Arangattuparambu Subcentre, Puthiyankandam, PHC-Melarkode, Ward-V Block-Pazhambalakode, District Palakkad .20 stool samplers were sent to Medical college Lab, Trichur. On culture 2 samples were confirmed for vibrio cholera 02 ogawa strain. |
| 46 | 2014 | Maharashtra | Sangli | Cholera | 36 | 0 | 11/8/2014 | 11/11/2014 | Under Control | Cases of diarrhea reported from Village-Vaddi, SC Dhavali, PHC-Mhaisal, Block Miraj,District Sangli. District RRT investigated the outbreak. House to house survey done. All cases treated symptomatically. Out of 04 stool sample tested at Sentinal Laboratory, Govt Medical college Miraj; 2 samples positive for Vibrio cholera by hanging drop & culture. Chlorination of water sources done.ORS packets distributed. Health education done regarding safe drinking water and sanitation. |
| 46 | 2014 | West Bengal | Hooghly | Cholera | 12 | 0 | 11/12/2014 | 11/14/2014 | Under Surveillance | Cases of loose watery stools with vomiting reported from Village Mosepukurpar, Subcentre Sarai, Adivasi Para District Hoogly. District RRT investigated the outbreak. Active case search done. 4 rectal swabs collected and tested sent to NICED; 3 positive for Vibrio Cholera 1 Ogawa. IEC given. Chlorination done. |
| 47 | 2014 | Bihar | Araria | Cholera | 20 | 4 | 9/8/2014 |  | Under Control | Cases of diarrhea reported from PHCs Katra, Sakra, Aurai and Musahari, District Muzzafarpur. District RRT investigated the outbreak. 5 samples sent to SKMCH, Muzzafarpur, result awaited... 4 positive for cholera on culture |
|  |  |  |  |  |  |  |  |  |  |  |
| Wk No. | Year | State | District | Disease | Cases | Deaths | Date of Outbreak | Date of Reporting | Current Status | Comments |
| 9 | 2015 | Andhra Pradesh | Kurnool | Cholera | 22 | 0 | 2/18/2015 | 2/19/2015 | Under Control | Cases reported from Village Adoni SC/ PHC Kosigi, District Kurnool. District RRT investigated the outbreak. House to House Survey done. Consumption of contaminated water due to leakages in the pipeline could be the probable cause of the outbreak. Alternate source of safe drinking water arranged. 07 stool samples tested positive for Vibrio and Salmonella. All cases treated symptomatically. Chlorination of water tanks done. Health education given. |
| 9 | 2015 | Gujarat | Ahemdabad | Cholera | 24 | 1 | 2/24/2015 | 2/24/2015 | Under Surveillance | Cases of vomiting and diarrhea reported from Village /SC Chandisar, PHC Ambaliyara, Block Dholka District Ahmedabad. District RRT investigated the outbreak. All the cases were residing in brick kiln area.02 water samples and 03 stool samples taken and sent to microbiology department of Medical College Ahmedabad; 02 stool samples were positive for cholera. Both the water samples were potable. One male aged 45 years died due to the condition. All cases treated symptomatically. Health education given. |
| 9 | 2015 | Telangana | Mahabubnagar | Cholera | 31 | 0 | 2/27/2015 | 2/27/2015 | Under Surveillance | Cases reported from Waddepally, District Mahabubnagar District RRT investigated the outbreak. Consumption of contaminated water due to leakages in the pipeline could be the probable cause of the outbreak. Active case search done. 05 stool samples were collected & send to DPL IDSP Mahabubnagar and 05 water samples collected and sent to IPM Lab Wanaparthy. Out of 5 stool samples, 1 tested positive for Vibrio, 1 for eneterococcus, and 1 for E.coli. 2 stool samples did not show any significant growth. All water samples were found to be potable. Alternate source of safe drinking water arranged. Medical camp organized in the area and all cases treated symptomatically. OHT cleaned and chlorination done. Health education given. |
| 16 | 2015 | Maharashtra | Raigad | Cholera | 22 | 0 | 3/30/2015 |  | Under Surveillance | Cases reported from SC Nigudshet, PHC Indapur, Taluka Mangaon, District Raigad. District RRT investigated the outbreak. House to house survey done. Consumption of contaminated water could be the probable cause of the outbreak.22 Sample Sent to DPHL Lab and 01 Sample found to be positive for cholera culture. All cases treated symptomatically. Health education given. |
| 17 | 2015 | Karnataka | Bidar | Cholera | 22 | 0 | 4/24/2015 | 4/25/2015 | Under Control | Cases with loose stools reported from Village Jojana, PHC Wadagaon, Taluk Aurad District Bidar. District RRT investigated the outbreak House to house survey done. Consumption of contaminated water from hand pump could be the probable cause of outbreak.01 out of 03 stool samples tested at Bidar Institute of Medical Sciences was found to be positive for cholera and 2 out of 06 water samples sent to DPHL were found to be non-potable. All the cases treated symptomatically. Chlorination done. Health education given. |
| 17 | 2015 | Karnataka | Kalaburagi | Cholera | 82 | 0 | 4/22/2015 | 4/23/2015 | Under Control | Cases reported from Village/PHC Biral B, Taluk Jewargi, District Kalaburagi. District RRT investigated the outbreak House to house survey done. Consumption of contaminated water from pond could be the probable cause of the outbreak. 01 out of 05 stool samples sent to Referral lab Bidar tested positive for cholera.16 water samples collected and sent for testing at DH; result awaited All cases treated symptomatically. Health education given. |
| 17 | 2015 | West Bengal | Nadia | Cholera | 59 | 0 | 4/15/2015 | 4/22/2015 | Under Control | Cases of fever with abdominal pain, vomiting, and loose motion reported from Village Bagalpur, GP Arbandi – II, Block Sanitpur, District Nadia. District RRT investigated the outbreak. House to house survey done.01 stool and 03 water sample sent to the Microbiology Lab, NICED at Beleghata. 01 out of 03 water samples was found to be positive for Vibrio Sp. Stool sample was found to be negative for any pathogen. All cases treated symptomatically. Health education given. |
| 19 | 2015 | Karnataka | Kalaburagi | Cholera | 53 | 0 | 5/5/2015 | 5/11/2015 | Under Control | Cases of loose stools reported from Village Melunda, PHC Hiresavalgi, Taluk,District Kalburgi. District RRT investigated the outbreak. Consumption of contaminated water due to leakages in drinking water pipeline could be the probable cause of outbreak. 06 water samples collected & sent to Referral Lab Bidar; result awaited. Out of 6 stool samples tested at Referal Lab Bidar, 1 stool sample was found positive for vibrio cholera on culture. All cases treated symptomatically. Pipeline repair done. Health education given. |
| 20 | 2015 | West Bengal | Hooghly | Cholera | 32 | 0 | 5/17/2015 | 5/18/2015 | Under Surveillance | Cases reported from Village Dahiya, GP Ektepur, Block Balagarh, and District Hooghly. District RRT investigated the outbreak. A total of 02 stool samples collected and sent to NICED Kolkata. One stool sample found positive for Cholera. All cases treated. Chlorination of water done. Health education given. |
| 20 | 2015 | West Bengal | Hooghly | Cholera | 29 | 0 | 5/11/2015 | 5/12/2015 | Under Surveillance | Cases reported from Village Inchura, GP Dhobapara, Block Balagarh, and District Hooghly. District RRT investigated the outbreak. A total of 02 stool samples collected and sent to NICED Kolkata. 2 water samples sent to BDO water testing lab. One stool sample found positive for Cholera. All cases treated symptomatically. Chlorination of water done. Health education given. |
| 22 | 2015 | Gujarat | Gandhinagar | Cholera | 9 | 0 | 5/25/2015 | 5/28/2015 | Under surveillance | Cases reported from Village Ghamij, PHC Bahiyal, Block Dehgam , District Gandhi Nagar. District RRT investigated the outbreak. 3 Stool sample collected and tested at GMERS Gandhinagar and out of that 2 samples were found to be positive for Vibrio Cholerae O1 Ogawa. All cases were treated symptomatically. Chlorination done.Health education regarding personal & environmental hygiene, regular chlorination of water given. |
| 22 | 2015 | Gujarat | Gandhinagar | Cholera | 1 | 0 | 5/25/2015 | 5/28/2015 | Under surveillance | Cases reported from Village Adlaj, PHC Sughad, Block/District Gandhi Nagar. District RRT investigated the outbreak. 1 Stool sample collected and tested at GMERS Gandhinagar and found positive for Vibrio Cholerae O1 Ogawa. All cases were treated symptomatically. Chlorination done. Health education regarding personal & environmental hygiene, regular chlorination of water given. |
| 22 | 2015 | Karnataka | Kalaburagi | Cholera | 17 | 0 | 5/25/2015 | 5/30/2015 | Under control | Cases reported from village/PHC Honnakarangi,Block/ District Kalburgi. District RRT investigated the outbreak. House to house survey done. One stool sample tested at DSU was found to be positive for cholera. All cases consumed well water for drinking purposes. Out of 15 water samples collected, 07 samples were found to be non-potable. Alternate water supply was provided. All cases treated symptomatically. Chlorination of water sources done. Health education given. |
| 23 | 2015 | Karnataka | Kalaburagi | Cholera | 22 | 1 | 4/7/2015 |  | Under control | Cases reported from Village Chinta Palli & Bootapura PHC Kodle Block Chincholi District Kalburgi. District RRT investigated the outbreak. House to house survey done.01 out of 06 stool sample tested at BRIMS Bidar, was found to be positive for cholera. All cases consumed well water for drinking purposes. Out of 15 water samples collected, 07 samples were found to be non-potable. One 5 year female child died due to the condition. Alternate water supply was provided. All cases treated symptomatically. Chlorination of water sources done. Health education given. |
| 24 | 2015 | Madhya Pradesh | Khargone | Cholera | 44 | 0 | 6/10/2015 | 6/12/2015 | Under Control | Cases reported from Village Bamkhal. CHC Kasrawad, Block Kasrawad, District Khargaon. District RRT investigated the outbreak. House to house survey done.Consumption of contaminated water could be the probable cause of the outbreak. A total of 02 stool and 02water samples were sent to Govt. Medical College Indore; one stool sample was found to be positive for V. Cholera Ogawa culture. Both the water sample were found to be potable. Water sources were chlorinated. All cases treated symptomatically. Health education given. |
| 25 | 2015 | Karnataka | Kalaburagi | Cholera | 156 | 0 | 6/16/2015 | 6/18/2015 | Under Control | Cases reported from Village /PHC Kalagi, Taluk Chittapur District Kalburgi. District RRT investigated the outbreak. Consumption of contaminated water from open well could be the probable cause of the outbreak.41 out of 70 water samples were found to be non-potable at DSU Kalburgi.14 stool samples sent to BRIMS, Bidar; all positive for cholera culture. Medical camp organized in the area and all cases treated symptomatically. Chlorination of water sources done. Alternate water supply organized. Health education given. |
| 26 | 2015 | Gujarat | Narmada | Cholera | 15 | 0 | 6/24/2015 | 6/25/2015 | Under Surveillance. | Cases reported from Village/SC/PHC Rajpipla, Taluka Nandod, District Narmada. District RRT investigated the outbreak. Consumption of contaminated water could be the probable causes of the outbreak.03 out of 10 stool samples tested at SSG Vadodara were found to be positive for cholera. All cases treated symptomatically. Health education given. |
| 26 | 2015 | Maharashtra | Nagpur | Cholera | 22 | 0 | 6/18/2015 | 6/24/2015 | Under Control | Cases reported from Haldiram factory, PHC Gumthala, Taluka Kamptee, District Nagpur. District RRT investigated the outbreak. House to house survey done. Consumption of contaminated water from factory well could be the probable cause of the outbreak.12 stool samples sent to GMC Nagpur out of which 10 samples were found to be positive for Cholera. Super chlorination done. All cases treated symptomatically. Health education given |
| 27 | 2015 | Karnataka | Belgaum | Cholera | 9 | 0 | 6/30/2015 | 7/1/2015 | Under Control | Cases reported from Village Dasarkodi, PHC/CHC Chinchali, Taluk Raibag, District Belgaum. District RRT investigated the outbreak. House to house survey done. 01 out of 2 stool samples tested positive for Vibrio Cholera culture at Belgaum Institute of Medical Sciences. All cases treated symptomatically. Health education given. |
| 27 | 2015 | Madhya Pradesh | Panna | Cholera | 56 | 2 | 7/1/2015 | 7/2/2015 | Under Surveillance | Cases reported from Gram Mudwari, Block Amanganj, District Panna.District RRT investigated the outbreak. House to house survey done. 01 out of 3 stool samples tested was found to be positive for vibrio cholera All cases treated Symptomatically. Health education given. |
| 28 | 2015 | Karnataka | Bidar | Cholera | 24 | 0 | 7/4/2015 | 7/7/2015 | Under Control | Cases reported from Village Narayanapura & Badalgaun Thanda, PHC/CHC Mudhol, Taluk Aurad, District Bidar. District RRT investigated the outbreak. House to house survey done. Consumption of contaminated water could be the probable cause of the outbreak.01 out of 2 stool samples tested at BIMS,Bidar was found to be positive for cholera culture. All cases treated symptomatically. Chlorination of water sources done. Health education given. |
| 30 | 2015 | Chandigarh | Chandigarh | Cholera | 2 | 0 | 7/20/2015 | 7/29/2015 | Under Surveillance | Cases of fever with rash reported from Manimajra, Sector-52 Chandigarh. District RRT investigated the outbreak. House to house survey done. Stool samples tested at District Priority Lab Manimajra, were found to be positive for Cholera. All cases treated symptomatically. Health education given. |
| 31 | 2015 | Chandigarh | Chandigarh | Cholera | 2 | 0 | 7/27/2015 | 8/5/2015 | Under Surveillance | Cases reported from Maulijagran area of Chandigarh. District RRT investigated the outbreak. House to house survey done. Stool samples sent to Department of Microbiology Govt. Medical College & Hospital Sector-32; 02 stool samples tested positive for Cholera culture. All cases treated symptomatically. Health education given. |
| 31 | 2015 | Karnataka | Bidar | Cholera | 15 | 0 | 7/27/2015 | 7/29/2015 | Under Control | Cases reported from Village Boral, PHC Changalera, Taluk Humnabad, District Bidar. District RRT investigated the outbreak. House to house survey done. Consumption of contaminated water could be the probable cause of the outbreak. 2 stool samples and 5 water samples tested at district Laboratory; One stool sample tested positive for cholera culture. All cases treated locally. Chlorination done. Health education given. |
| 31 | 2015 | Punjab | Ludhiana | Cholera | 414 | 4 | 8/3/2015 | 8/4/2015 | Under Surveillance | Cases reported Baba Jeewan Singh Nagar, Tajpur Road, Ludhiana. District RRT investigated the outbreak. House to house survey done.02 out of 08 stool samples tested at CMC Ludhiana were found to be positive for cholera culture. 23 water samples tested at CMC Ludhiana; out of which 10 samples were found to non-potable.02 females aged 3years and 60 years and 02 males aged 6years and 70 years respectively died due to the condition. All cases treated symptomatically. Health education given. |
| 32 | 2015 | Chandigarh | Chandigarh | Cholera | 16 | 0 | 8/8/2015 | 8/13/2015 | Under Surveillance | Cases reported from Indira Colony Manimajra and Maulijagran. District and state RRT investigated the outbreak. House to house survey done. Stool samples sent to Department of Microbiology Govt. Medical College & Hospital Sector-32; 08 stool samples tested positive for Cholera culture. All cases treated symptomatically. Health education given. |
| 33 | 2015 | Dadra & Nagar Haveli | Dadra & Nagar Haveli | Cholera | 1 | 0 | 8/14/2015 | 8/14/2015 | Under Surveillance | Cases of loose stools reported from Village/SC Athal, PHC Naroli, Block Silvassa. District RRT investigated the outbreak.01 Stool sample tested at DPHL, Silvassa was found to be positive for V. Cholerae O1. Cases treated symptomatically. Health education given. |
| 33 | 2015 | Madhya Pradesh | Ratlam | Cholera | 878 | 0 | 7/2/2015 |  | Under Control | Cases reported from 03 Villages namely Kelkacch, Raoti and Ratangarhpeeth, Block Bajana. District RRT investigated the outbreak. House to house survey done. Consumption of contaminated water could be the probable causes of the outbreak.04 out of 17 stool samples tested at MGM College Indore were found to be positive for Vibrio Cholerae culture. All cases treated symptomatically. Health education given. |
| 34 | 2015 | Dadra & Nagar Haveli | Dadra & Nagar Haveli | Cholera | 2 | 0 | 8/22/2015 | 8/22/2015 | Under surveillance | Cases reported from Village / Sub Center/PHC Masat& Village/SC Sayli, PHC Rakholi Block Silvassa. 02 out of 04 stool samples tested at DPHL Silvassa were found positive for Vibrio Chlorea O1 ogawa.01 out of 02 water samples DPHL, Silvassa was found to be non potable.All cases treated symptomatically. Health education given. |
| 34 | 2015 | West Bengal | Bankura | Cholera | 34 | 0 | 8/17/2015 | 8/21/2015 | Under surveillance | Cases reported from Village/SC Ethani, GP Jhunjka Block Chhatna,.District RRT investigated the outbreak. House to house survey done. Consumption of contaminated water could be the probable cause of the outbreak. All 07 water samples tested at PHE Laboratory were found to be non potable.01out of 02 stool samples tested at BS Medical College Bankura was found to be positive for Vibrio Cholera 01 Ogawa. All cases treated symptomatically. Health education given. |
| 34 | 2015 | Madhya Pradesh | Indore | Cholera | 6 | 0 | 7/27/2015 |  | Under Control | Cases reported from different areas of Indore city. District RRT investigated the outbreak. House to house survey done.06 samples tested positive for V. cholera Ogava grown at MGM Medical college, Indore. All cases treated symptomatically. Health education given. |
| 35 | 2015 | Karnataka | Udupi | Cholera | 7 | 2 | 8/22/2015 | 8/26/2015 | Under Surveillance | Cases reported from Village Thenkanidiyooru, PHC/CHC Kemmannu, Taluk Udupi. District RRT investigated the outbreak. House to house survey done. 03 samples tested positive for vibrio Cholera Eltor Serotype Ogawa in culture. Two males aged 38 years and 45 years respectively died due to the condition. All cases treated symptomatically. Health education given. |
| 35 | 2015 | Punjab | Hoshiarpur | Cholera | 18 | 0 | 8/26/2015 | 8/26/2015 | Under Surveillance | Cases reported from Village Badhan S/C Sehrlowal, PHC Budhawar, Block Mukerian, District RRT Investigated the outbreak. House to house survey done. 07 stool samples tested at DPHL Hoshiyarpur were found to be positive for V.Cholera Ogava. All cases treated symptomatically. Health education given. |
| 35 | 2015 | Madhya Pradesh | Indore | Cholera | 5 | 0 | 8/17/2015 |  | Under Control | Cases reported from Vinoba Nagar Palaysia, Dwarkapuri, TakshilaParisar Indore. District RRT investigated the outbreak. House to house survey done. Pipeline leakages were observed in the community. Consumption of contaminated water could be the probable cause of the outbreak. All 05 stool samples tested at MGM Medical college, Indore were found to be positive cholera culture. All cases treated symptomatically. Health education given. |
| 35 | 2015 | Madhya Pradesh | Indore | Cholera | 3 | 0 | 8/10/2015 |  | Under Surveillance | Cases reported from 1 Ahilyamata Colony, Y.N. Road. Dwarkapuri, Indore. District RRT investigated the outbreak. House to house survey done. Pipeline leakages were observed in the community. Consumption of contaminated water could be the probable cause of the outbreak. All 03 stool samples tested at MGM Medical College, Indore were found to be positive cholera culture. All cases treated symptomatically. Health education given. |
| 36 | 2015 | West Bengal | Bankura | Cholera | 62 | 1 | 8/29/2015 | 9/1/2015 | Under surveillance | Cases reported from Village Adibasipara, SC Hatibari, GP Dubrajpur, Block Simlapal. District RRT Investigated the outbreak. House to house survey done. 02 out of 05 water samples tested at Bankura water testing laboratory were found to be non potable. 01 out of 03 stool samples tested at BS MC Bankura was found to be positive for V .Cholerae Ogawa. One 55 year old female died due to the condition. All cases treated symptomatically. Health education given. |
| 36 | 2015 | Rajasthan | Baran | Cholera | 55 | 0 | 8/20/2015 |  | Under Surveillance | Cases reported from Village Chhatraganj, Block Kishanganj, PHC Nahargarh. District RRT investigated the outbreak. House to house survey done. 03 stool samples and 02 water samples collected and sent to central micro biology laboratory MBS Kota. 01 out of 03 stool samples tested positive for cholera culture. 02 water samples were found to be non potable.All cases treated symptomatically. Health education given. |
| 39 | 2015 | Chandigarh | Chandigarh | Cholera | 3 | 0 | 9/19/2015 | 9/30/2015 | Under Surveillance | Cases reported from Vikas Nagar Maulijagran. District RRT investigated the outbreak. House to house survey done. 02 stool samples tested at DPL Manimajra found to be positive for Vibrio Cholera (Ogawa O1 ) and 01 stool sample tested at PGIMER Chandigarh was found to be culture positive for cholera. All cases treated symptomatically. Chlorination of water source done. Health education given. |
| 39 | 2015 | Karnataka | Bagalkot | Cholera | 66 | 0 | 9/20/2019 | 9/25/2019 | Under Control | Cases reported from Village/PHC/CHC Kandgal, Taluk Hunagund, District RRT investigated the outbreak. House to house survey done. Pipeline leakages were observed in the community. Consumption of contaminated water could be the probable cause of the water. 01 stool sample tested positive for cholera culture at BIMS Belgaum. 03 Out of 08 water samples tested at district Hospital Laboratory were found to be non potable. All cases treated symptomatically. Health education given. |
| 39 | 2015 | West Bengal | Purulia | Cholera | 15 | 0 | 9/1/2015 |  | Under Surveillance | Cases reported from Village/SC Puiara, Block Barabazar. District RRT investigated the outbreak. House to house survey done. 01 Out of 02 rectal swab tested, was found to be positive for vibrio cholera 01 Ogawa culture. All cases treated symptomatically. Health education given. |
| 40 | 2015 | Telangana | Mahabubnagar | Cholera | 172 | 1 | 9/28/2015 | 10/5/2015 | Under Control | Cases reported from Village Nagardoddy, SC Thatikunta, PHC/ Mandal Maldakal. District. RRT investigated the outbreak. House to house survey done. 01 out of 03 stool samples tested at District Priority Laboratory Mahbubnagar was found to be positive for cholera. 06 out of 09 Water samples tested at IPM Laboratory, Wanaparthy were found to be non-potable. One 60 year old female died due to the condition.All cases treated symptomatically. Health education given. |
| 41 | 2015 | Gujarat | Anand | Cholera | 72 | 1 | 10/6/2015 | 10/7/2015 | Under Surveillance | Cases reported from Village/UHC/SC Borsad, Block Ankla. District RRT investigated the outbreak. House to house survey done.pipeline leakages were observed in the community. 02 out of 05 stool samples tested positive for cholera. 03 Water samples sent to DPHL ; result awaited. Chlorination of water sources done. One 86 year old female died due to the condition. All cases treated symptomatically. Health education was given. |
| 47 | 2015 | Karnataka | Mysore | Cholera | 65 | 0 | 11/20/2015 | 11/20/2015 | Under Control | Cases reported from Village/Taluk Mysuru City, PHC/CHC Shanthinagar, Kyathamaranalli, HHMBG, GB Palya, NR Mohalla. District RRT investigated the outbreak. House to house survey done. Consumption of contaminated water could be the probable cause of the outbreak. 25 Water samples tested at DSU laboratory Mysuru out of which 17 samples were found to be non-potable. All 03 Stool samples tested at Cheluvamba Hospital were found to be positive for cholera. All cases treated symptomatically. Health education given. |
| 49 | 2015 | West Bengal | Purulia | Cholera | 31 | 0 | 11/29/2015 | 11/30/2015 | Under Surveillance | Cases reported from Village Ramer Bagicha, SC Sanka, Block Raghunathpur-I. District RRT investigated the outbreak. House to house survey done. Drinking of contaminated water could be the probable cause of the outbreak. 02 Rectal swabs tested at PHE Laboratory Purulia. Both rectal samples were found to be positive for Vibrio cholera culture. All cases treated symptomatically. Health education given. |
| 51 | 2015 | Rajasthan | Jaipur | Cholera | 7 | 0 | 12/14/2015 | 12/22/2015 | Under Control | Cases reported from Village Khushar, PHC Beelwa, BlockSanganer. District RRT investigated the outbreak. House to house survey done. Poor hygiene and water through bore well could be the probable cause of the outbreak. 01Rectal swab tested at SMS Medical College & Hospital was found to be positive for V. Cholerae. 01 Water sample collected; result awaited. ORS packets distributed. All cases treated symptomatically. Health education given. |
